# The Chromosome-Scale Reference Genome of *Macadamia tetraphylla* Provides Insights Into Fatty Acid Biosynthesis

**DOI:** 10.3389/fgene.2022.835363

**Published:** 2022-02-23

**Authors:** Yingfeng Niu, Guohua Li, Shubang Ni, Xiyong He, Cheng Zheng, Ziyan Liu, Lidan Gong, Guanghong Kong, Wei Li, Jin Liu

**Affiliations:** ^1^ Yunnan Institute of Tropical Crops, Xishuangbanna, China; ^2^ School of Life Sciences, Institute of Life Sciences and Green Development, Hebei University, Baoding, China

**Keywords:** *Macadamia tetraphylla*, nanopore sequencing, Hi-C, whole genome duplication, fatty acid biosynthesis

## Abstract

Macadamia is an evergreen tree belonging to the Proteaceae family. The two commercial macadamia species, *Macadamia integrifolia* and *M. tetraphylla*, are highly prized for their edible kernels. The *M. integrifolia* genome was recently sequenced, but the genome of *M. tetraphylla* has to date not been published, which limits the study of biological research and breeding in this species. This study reports a high-quality genome sequence of *M. tetraphylla* based on the Oxford Nanopore Technologies technology and high-throughput chromosome conformation capture techniques (Hi-C). An assembly of 750.87 Mb with 51.11 Mb N50 length was generated, close to the 740 and 758 Mb size estimates by flow cytometry and k-mer analysis, respectively. Genome annotation indicated that 61.42% of the genome is composed of repetitive sequences and 34.95% is composed of long terminal repeat retrotransposons. Up to 31,571 protein-coding genes were predicted, of which 92.59% were functionally annotated. The average gene length was 6,055 bp. Comparative genome analysis revealed that the gene families associated with defense response, lipid transport, steroid biosynthesis, triglyceride lipase activity, and fatty acid metabolism are expanded in the *M. tetraphylla* genome. The distribution of fourfold synonymous third-codon transversion showed a recent whole-genome duplication event in *M. tetraphylla*. Genomic and transcriptomic analysis identified 187 genes encoding 33 crucial oil biosynthesis enzymes, depicting a comprehensive map of macadamia lipid biosynthesis. Besides, the 55 identified *WRKY* genes exhibited preferential expression in root as compared to that in other tissues. The genome sequence of *M. tetraphylla* provides novel insights for breeding novel varieties and genetic improvement of agronomic traits.

## Introduction

Macadamia is an evergreen nut tree belonging to the Proteaceae family, genus *Macadamia* F. Muell, commercially grown for their high-value kernels (Toft et al., 2018). The genus *Macadamia* F. Muell. contains four different species, namely, *M. integrifolia*, *M. tetraphylla*, *M. ternifolia*, and *M. jansenii* (Akinsanmi et al., 2017), but only *M. integrifolia*, *M. tetraphylla*, and their hybrids (*M*. *integrifolia* × *M*. *tetraphylla*) are used for commercial nuts production (Hardner, 2016). Due to the large-scale commercial cultivation in Hawaii (United States) in 1948 (Ahmad Termizi et al., 2014), macadamia was also named as Hawaiian Nuts, which is native to the subtropical rainforest of Queensland, New South Wales, Australia (Neal et al., 2010; Shapcott and Powell, 2011).

Macadamia kernels are rich in unsaturated fatty acids, essential amino acids, trace elements, and vitamins, but monounsaturated fatty acids and palmitoleic acid contents are extremely high (Kaijser et al., 2000). Macadamia nuts are eaten directly or used as raw material for processing high-grade edible oil (Navarro and Rodrigues, 2018). Macadamia nuts are very nutritious and deeply subjected to a large consumer approbation fancy, to whom it is also known as the “Queen of nuts.” Long-term consumption of macadamia lowers blood cholesterol and viscosity of platelets, prevents arteriosclerosis, reduces heart disease, myocardial infarction, and other cardiovascular diseases (Garg et al., 2003; Garg et al., 2007), World consumption of macadamia nuts has rapidly grown in recent years with more than 400,000 tons in demand (FAO statistics, year), yet the current global supply is only ∼40,000 tons. Within the current and future periods, macadamia nuts production is still in short supply (Trueman and Turnbull, 1994). The commercial growing areas of macadamia are located in the tropical and subtropical belts, although the traditional cultivated area is in the United States and Australia (Trueman and Turnbull, 1994). The total macadamia planted area has rapidly grown over the past decade because the cultivation technology is simple, planting high income, the plants are more cold-resistant than rubber trees, bananas, and other traditional tropical crops, yet very suitable for the cool weather of tropical and subtropical countries. Globally, the largest macadamia orchard area is in China (300,000 ha), followed by South Africa, Australia, Kenya, Guatemala, and the United States in that order.

Macadamia is diploid (2n = 28) with genome size estimates of 652–780 Mb (Chagné, 2015). In recent years, the genome sequences of many important tropical crops have been reported, but reports on the genome of macadamia are very few. The chloroplast genomes of *M. integrifolia, M. ternifolia,* and *M. tetraphylla* were sequenced in 2014, 2017, and 2018 by Australian and Chinese researchers (Nock et al., 2014; Liu et al., 2017; Liu et al., 2018), and the draft genome and transcriptome of *M. integrifolia* cultivar 741 was sequenced in 2016. The total assembly length is 518 Mb, spanning ∼79% of the estimated genome size (Nock et al., 2016). However, there remains no report on the genome sequencing of *M. tetraphylla* to date. *M. tetraphylla* is an important parent species of commercially grown macadamia varieties (Pisanu et al., 2009), and the genome sequencing of *M*. *tetraphylla* will provide abundant genetic information and references for the screening of breeding materials.

## Materials and Methods

### Sample Collection, Library Construction, and Sequencing

A cultivated *M. tetraphylla* plant was collected from Xishuangbanna, Yunnan Province, China. The collected plant samples were immediately frozen in liquid nitrogen and stored at −80°C before DNA isolation. High-molecular-weight genomic DNA was extracted using a Qiagen plant genomic DNA extraction kit (QIAGEN, Hilden, Germany). Sequencing library was prepared with Nanopore Genomic Sequencing Kit SQK-MAP006 (ONT, United Kingdom) and a PCR-free “native barcoding” kit provided by ONT. Blunt/TA ligase Master Mix (M0367S, NEB) was used to ligate native barcode adapters for 10 min at room temperature. A 1:1 volume of AMPure XP beads was used to purify the barcoded DNA, and the DNA was eluted in 26 μL nuclease free water. Hairpin adapters were ligated and added to the pooled library DNA to give a final reaction volume of 100 μL. The reaction mixture was incubated for 10 min at room temperature. The final reaction was cleaned using prewashed Dynabeads MyOne Streptavidin C1 beads (65001; Thermo Fisher Scientific). The library was loaded on a single R9.4 flow cell and sequenced on a GridION X5 platform (Oxford Nanopore Technology, OX4 4DQ, Oxford, United Kingdom). Reads were trimmed according to a minimum read quality of Q15. Reads with length shorter than 30 bp were excluded and sequencing adapters were removed. MinION (TM) 2D reads were filtered into passed and failed reads using Metrichore basecaller.

To construct the Hi-C library, ∼4–6 g young leaves were harvested and frozen in liquid nitrogen. The nuclear DNA was cross-linked *in situ* in 2% formaldehyde at room temperature before extraction. The nuclei extracted were then restricted with HindIII restriction enzyme. Free ends were then biotinylated, diluted and ligated randomly. The generated libraries were sequenced on Illumina Hiseq4000 platform.

Total RNA was isolated from five tissues (young leaves, young inflorescences, flowering inflorescences, proteoid roots, and barks) using the Column Plant RNAout kit (TIANDZ, Beijing, China). A 300-cycle kit was used for a 2 × 150 bp paired-end run and the generated library was sequenced on the Illumina HiSeq2500 platform.

### Estimation of Genome Size and Heterozygosity

The *M. tetraphylla* genome size was estimated by flow cytometry following the protocol described by Dolezel (Doležel et al., 2007) and k-mer frequency analysis. In brief, Jellyfish v2.1.0 (Marçais and Kingsford, 2011) was used to generate the 17-mer frequency distribution of paired-end reads. The genome size was estimated according to the formula: G = K_num/peak depth (G: genome size; K_num: total number of k-mers; peak depth: depth of the major peak). The GenomeScope (Vurture et al., 2017) estimated heterozygosity of the *M. tetraphylla* genome is 1.03%.

### Genome Assembly

*De novo* assembly pipeline was used to alleviate the dual effects of the highly heterozygous genome and highly repetitive DNA sequences. The Nanopore raw reads were corrected and trimmed using the Canu v1.8 (Koren et al., 2017) software. The corrected reads were uploaded to the WTDBG v2.2 (Ruan and Li, 2020) for genome assembly using the following parameters: S 2 --edge-min 2 --rescue-low-cov-edges -x ccs -g 800 m. Iterative polishing was performed using the Pilon v1.23 (Walker et al., 2014) software with the Illumina genomic data to fix bases, fill gaps, and correct local misassemblies. The polished contigs were subsequently processed by the 3d-DNA pipeline (version 170123) (Dudchenko et al., 2017) using default parameters. Juicebox (https://github.com/aidenlab/Juicebox) was used to visualize the resulting Hi-C contact matrix, which was manually corrected based on the neighboring interactions. A total of 14 high-confidence clusters were identified in the *M. tetraphylla* genome. A visualization of the assembly contiguity was generated using assembly-stats (https://github.com/rjchallis/assembly-stats).

The Illumina sequencing reads were mapped to the genome using bowtie2 v2.2.6 (Langmead and Salzberg, 2012) to assess the completeness and accuracy of the genome assembly. Additionally, the assembly was evaluated by BUSCO (Benchmarking Universal SingleCopy Orthologs) (Simão et al., 2015). To further evaluate the genome assembly, the RNA reads were mapped to the genome using HISAT2 (Daehwan et al., 2015). The GC content was calculated with a 2 kb non-overlapping sliding window. To assess the accuracy of the genome assembly, we also mapped the ONT long reads to the genome using minimap2 v2.17-r941 (Li, 2018) with the “map-ont” option.

### Repeat Annotation

Two complementary methods were used to identify repetitive sequences in the *M. tetraphylla* genome. First, the Tandem Repeats Finder v4.09 (Benson, 1999) was employed to identify the tandem repeats. Second, a combined strategy was selected to predict transposable elements (TEs). For the homology-based annotation of TEs, RepeatMasker v1.332 (http://www.repeatmasker.org) was employed to search the RepBase database (v18.07) (Bao et al., 2015) for repetitive DNA, and RepeatProteinMasker (Tarailo-Graovac and Chen, 2009) to search the protein database for TE-related proteins. A *de novo* library was constructed using three software, including RepeatModeler v1.05 (http://www.repeatmasker.org/RepeatModeler.html), RepeatScout v1.05 (Price et al., 2005), and Piler v1.06 (Edgar and Myers, 2005). The RepeatMasker was then applied to identify TEs comprehensively.

Simple sequence repeats (SSRs) in the *M. tetraphylla* genome were identified using the MISA program (Thiel et al., 2003) with the following parameters: at least twelve repeats for monomer, six repeats for the dimer, four repeats for trimer, three repeats for tetramer, pentamer, and hexamer.

### Gene Prediction and Functional Annotation

The Augustus v2.7 (Mario et al., 2004) and SNAP v2006-07-28 (Korf, 2004) software were used to perform *de novo* prediction. Genes were predicted from the combination of *de novo*, homology, and EST predictions. The assembled transcripts were used for iterative self-training, and the optimized parameters were applied for further annotation by the Augustus and SNAP software. For homology prediction, protein sequences from *Arabidopsis thaliana* (Initiative, 2000), *Malus domestica* (Velasco et al., 2010), *Nelumbo nucifera* (Ming et al., 2013), and *Rosa chinensis* (Saint-Oyant et al., 2018) were aligned to the genome using the genblastA v1.0.1 (She et al., 2009) software. The homologous genomic regions of the target genes were extended in both 3′ and 5′ directions and then loaded to the GeneWise v2.2.0 (Birney et al., 2004) software to obtain accurate spliced alignments. The transcripts were also mapped to the genome to generate spliced alignments using the Program to Assemble Spliced Alignments (PASA) pipeline (version 2.0.2) (Haas et al., 2003). Finally, all these predictions were consolidated into a consensus gene set using EVidenceModeler (r2012-06-25) (Haas et al., 2008).

Functional assignment was performed using BLASTP (with 1e-5 e-value) to search against the SwissProt database (Bairoch and Apweiler, 2000). The KAAS server (Yuki et al., 2007) was used to map the predicted genes onto KEGG metabolic pathways. InterProScan v5.10-50.0 (Jones et al., 2014) software determined the motifs and functional domains. The GO term and Pfam domains were directly obtained from the InterProScan results.

The *Arabidopsis* gene (TAIR10) was used to search for genes related to the oil biosynthesis pathway (Supplementary Table S1). Target genes with ≥50% sequence coverage in length and functional annotations were classified into corresponding gene families. The identified genes were further refined by searching the Pfam database.

### Noncoding RNA Annotation

Five types of noncoding RNA genes, tRNA, rRNA, snRNA, snoRNA, and miRNA, were identified in the *M. tetraphylla* genome. The tRNA genes were identified using the tRNAscan-SE v2.0 (Schattner et al., 2005) software with default parameters. The RNAmmer v1.2 (Lagesen et al., 2007) software was performed to predict rRNAs and their subunits with default parameters. The snRNA genes were predicted using the INFERNAL software (v1.1.2) (Nawrocki et al., 2009) with cm models from the Rfam database (Griffiths-Jones et al., 2005). The snoRNA genes were identified by the snoscan v0.9.1 (Lowe and Eddy, 1999) software.

### Comparative Genome Analyses

Orthologous gene clusters were computed using the OrthoMCL pipeline (Li et al., 2003) with the following finished genomes: *Actinidia chinensis*, *Coffea canephora*, *Populus trichocarpa*, *Oryza sativa*, *A*. *thaliana*, *R*. *chinensis*, *N*. *nucifera*, and *M*. *tetraphylla*. An all-against-all comparison was performed using the BLASTP search tool with a 1e-5 e-value. For each single-copy gene family, protein sequences were aligned by the MUSCLE software (Edgar, 2004) and subsequently joined into one supergene for each species. The phylogenetic tree was inferred using the RAxML software (Stamatakis, 2014) with PROTGAMMAWAG model and 1000 bootstraps. The CAFE (Computational Analysis of Gene Family Evolution) tool (De Bie et al., 2006) was used to detect gene family expansion and contraction with a probabilistic graphical model. The GSEA (Gene Set Enrichment Analysis) was performed with the Fisher’s exact test (*p*-value < 0.05) on InterPro domains.

To further analyze the major evolutionary events in *M. tetraphylla*, the 4DTv (fourfold synonymous third-codon transversion) distribution in the *M. tetraphylla* genome was calculated. The two proteomes were compared using BLASTP (1e-5 e-value). Syntenic blocks were identified using the MCscanX toolkit (Wang et al., 2012) with <5 intervening genes between hits. The 4DTv between gene pairs located in synteny blocks were calculated using an in-house Perl script.

### RNA-Seq Data Analysis

The raw reads of RNA-seq data were trimmed using Trimmomatic v0.32 (Bolger et al., 2014) to improve the quality. The adaptors and low-quality reads with <15 average quality per base and a 4-base wide sliding window were trimmed off. And then, the resulting clean data were aligned to the reference genome using the HISAT2 (Kim et al., 2015) software. The FPKM expression of target genes was calculated using the Stringtie (Pertea et al., 2016) transcript assembler.

## Results

### Genome Sequencing and Assembly

**FIGURE 1:**
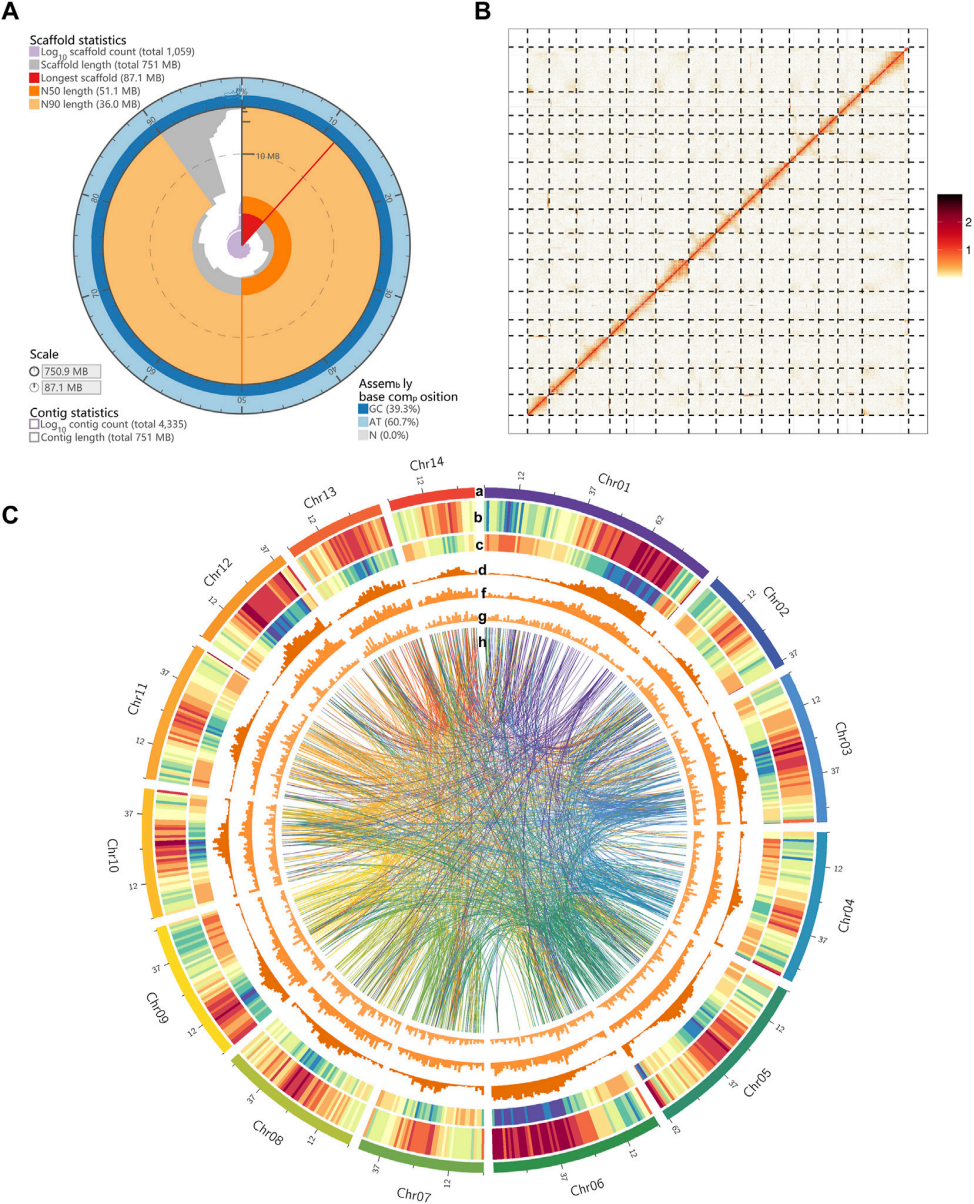
Landscape of macadimia genome. **(A)** Visualization of assembly stats (https://github.com/rjchallis/assembly-stats): the inner radius (highligthed in red color) represents the length of the longest scaffold, the radial axis originates at the circumference indicates the scaffold length, the N50 and N90 scaffold lengths are indicated respectively by dark and light orange arcs, respectively. The cumulative number of scaffolds within a given percentge of the genome is plotted in purple. The outermost circular layer shows the base composition at the given coverage of the genome. **(B)** Hic-contact map of macadimia genome. **(C)** Circos plot of macadimia genome. Tracks from outside to inside are the 14 chromosomes of *M. tetraphylla*, gene density (density measured in 1000-Kb sliding windows), transposable element (TE) density, *Gypsy*-type LTR retrotransposons density, *Copia*-type LTR retrotransposons density, DNA transposable element density. The syntenic blocks within chromosomes of macadimia genome are displayed with connecting lines in different colors.

The *M. tetraphylla* was sequenced on the Oxford Nanopore Technologies (ONT) platform. A total of 68.17 Gb of data were generated with an average read length of 20.16 kb (Supplementary Table S2 and Supplementary Figure S1A) from the Nanopore platform, and 88.27 Gb of short reads from the HiSeq2500 platform (Illumina, CA, United States) with 500 bp insert size to genome survey and assembly polishing (Supplementary Table S3). After correcting, a total of 26.34 Gb clean data was obtained with an average length of 29.89 kb (Supplementary Figure S1B). The *M. tetraphylla* genome size was estimated by flow cytometry, and the deduced genome size was 740 Mb. We also evaluated the genome size using k-mer analysis. The cumulative k-mer count suggested a 758 Mb genome size (Supplementary Figure S2), similar to the flow cytometry results. A *de novo* assembly pipeline was used to alleviate the dual effects of the highly heterozygous genome and highly repetitive DNA sequences (Supplementary Figure S3). Finally, the *M. tetraphylla* genome was assembled into 4,335 contigs, with an N50 of 1,182,547 bp (Table 1). The assembly size (750.87 Mb) is consistent with the estimated genome size based on flow cytometry and k-mer analysis (740 and 758 Mb, respectively). The N50 contig showed a 335.8-fold increase compared to *M. integrifolia* (Figure 1A and Supplementary Table S4). Two Hi-C libraries were constructed from young leaves of *M. tetraphylla*. About 533 million 150-bp paired-end Illumina reads were produced and uniquely mapped onto the draft assembly contigs. The insertion size of Hi-C reads spanned predominantly from dozens to hundreds of kilobases, therefore providing efficient information for scaffolding (Figure 1B and Supplementary Table S5). Notably, 95.29% (715.17 Mb) of the genome anchored to 14 pseudo-chromosomes combined with the valid Hi-C data (Figure 1C, Supplementary Figure S4 and Supplementary Table S5). The chromosome lengths varied from ∼30.93 to ∼87.10 Mb with ∼51.11 Mb N50 size (Table 1, Supplementary Table S6).

**TABLE 1:** Summary of the *M. tetraphylla* genome assembly and annotation.

Assembly	
Sequencing Depth (×)	89.93
Estimated genome size (Mb)	758
Assembled sequence length (Mb)	750.54
Scaffold N50 (bp)	51,109,939
Contig N50 (bp)	1,182,547
Annotation,	
Number of predicted protein-coding genes	31,571
Average gene length (bp)	6,055
tRNAs	1,286
rRNAs	542
snoRNAs	74
snRNAs	251
Transposable elements (%)	61.42

Quality-filtered Illumina reads were mapped to the genome to validate the completeness and accuracy of the genome assembly (Supplementary Figure S5). Results showed that 94.25% of the short reads mapped to the genome, with an 87.84% properly-paired mapping rate (Supplementary Table S7). The accuracy and completeness of the assembly were also assessed by mapping the ONT long reads to the genome. Overall mapping rate of these long reads was 99.81% for our assembly (Supplementary Table S7). From the BUSCO software, ∼89.72% (1,292 out of 1,440) conserved genes in the embryophyta lineage were present in the assembly (Supplementary Table S7). Additionally, 92.00% of the RNA-Seq data independently aligned to the assembled genome. We have also calculated the GC content with a 2 kb non-overlapping sliding window established no obvious GC bias in the genome assembly (Supplementary Figure S5). Altogether, these results suggest a high-quality genome of *M. tetraphylla*.

### Repeats and Gene Annotation

We identified 461 Mb of repetitive sequences, accounting for 61.42% of the genome. These repetitive sequences mainly comprised transposable elements, including RNA retrotransposons (Class I) and DNA transposons (Class II). Long terminal repeat (LTR) retrotransposons represent the most predominant class of transposable elements. The assembled *M. tetraphylla* contains 34.95% LTR retrotransposons, of which 22.00% are *Gypsy*-type elements, and 5.94% are *Copia*-type elements (Supplementary Table S8). A total of 510,893 SSRs were also identified in the *M. tetraphylla* genome (Supplementary Table S9). Among the repeat motifs, mono-nucleotide repeats were the most predominant, followed by di-, tri-, tetra-, penta-, and hexa-nucleotide (Supplementary Figure S6 and Supplementary Table S9). The identified SSR markers may serve as potential markers to *M. tetraphylla* breeding programs.

A total of 34.30 Gb RNA-seq data were obtained from five tissues representing major tissue types and developmental stages (Supplementary Table S3) to further aid in gene prediction. The 31,571 genes identified combine *de novo*, homology-based and EST-based evidence. The average length of the identified genes, exons, and introns were 6,055, 222, and 1,213 bp, respectively (Table 1 and Supplementary Table S10). Overall, 29,233 genes (92.59%) were functionally assigned to the public database, of which 22,869 (72.44%) genes had Swiss-Prot homologs, 8,303 (26.30%) had KEGG homologs, 29,052 (92.02%) had InterPro homologs, 17,864 (56.58%) had GO homologs and 21,925 (69.45%) had Pfam homologs (Supplementary Figure S7 and Supplementary Table S11). Up to 1,286 tRNAs, 542 rRNAs, 251 snRNAs, and 74 snoRNAs were identified in the *M. tetraphylla* genome (Supplementary Table S12).

### Expanded Gene Families and Whole-Genome Duplication

**FIGURE 2:**
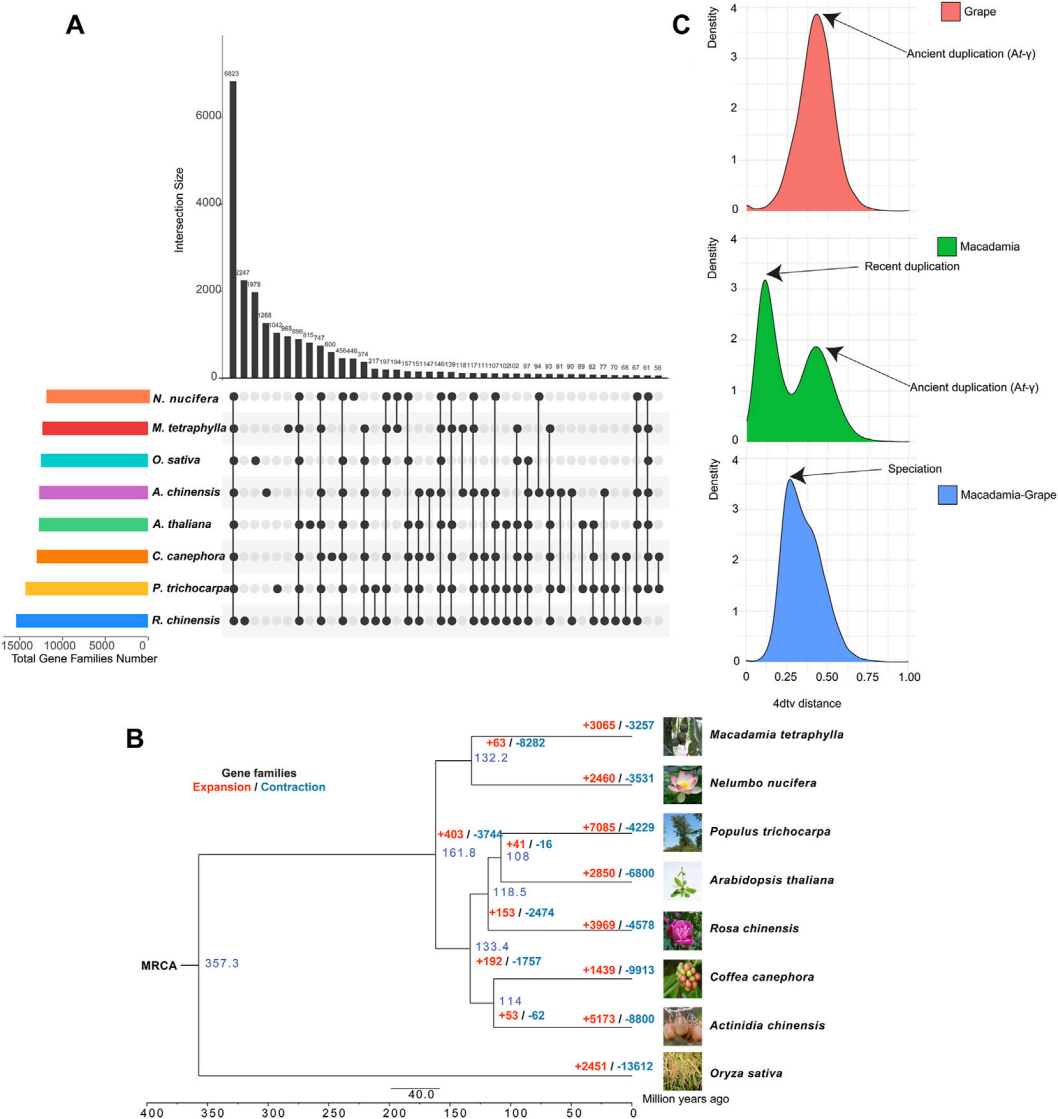
Evolution of macadimia genome. **(A)** Venn diagram showing shared and unique gene families among macadimia and other plant species. **(B)** Comparative genomic analysis of macadimia and other plant species. **(C)** Distribution of 4DTv for pairs of syntenic paralogs.

The sequenced *M. tetraphylla* genome was further compared with seven other sequenced plant genomes, including *A. chinensis*, *A*. *thaliana*, *C*. *canephora*, *N*. *nucifera*, *O*. *sativa*, *P*. *trichocarpa*, and *R*. *chinensis,* to investigate the genetic basis underpinning the distinct traits of *M. tetraphylla*. A total of 24,346 orthologous gene families consisting of 204,948 genes were identified (Supplementary Figure S8 and Supplementary Table S13). The eight plant species shared a core set of 107,264 genes which belong to 6,823 gene families and represent ancestral gene families (Figure 2A and Supplementary Table S13). Besides, 965 gene families containing 4,337 genes were unique to *M. tetraphylla* (Figure 2A and Supplementary Table S13).

The GO annotation revealed that the *M. tetraphylla* specific gene families are highly enriched for the chitin catabolic process (GO:0006032, *p* < 1.23E-02), polysaccharide binding (GO:0030247, *p* < 3.99E-07), and ADP binding (GO:0043531, *p* < 5.94E-08). The *M. tetraphylla* GO enrichment is probably related to the thick shells of macadamia nuts (Supplementary Figure S9 and Supplementary Table S14). The *M. tetraphylla* specific gene families also mapped to the KEGG pathways of carbohydrate metabolism, metabolism of other amino acids, biosynthesis of other secondary metabolites, xenobiotics biodegradation, metabolism, and protein families for genetic information processing, signaling, and cellular processes (Supplementary Figure S10).

A phylogenomic tree constructed using 833 single-copy genes from the eight plant species showed that *M. tetraphylla* is closely related to *N. nucifera* but diverged ∼132.2 million years ago (MYA) (Supplementary Figure S11). The gene families with significant change are often associated with distinct traits (Zhang et al., 2017). A gene family evolutionary analysis of the eight plant species showed that 3,065 gene families underwent expansion, whereas 3,257 underwent contraction (Figure 2B, Supplementary Figures S12A,B).

The GO enrichment analysis revealed that the expansion of these families involved genes related to defense response (GO:0042742, GO:0006952), lipid transport (GO:0006869), steroid biosynthetic process (GO:0006694), triglyceride lipase activity (GO:0004806), and fatty acid metabolic process (GO:0006631) (Supplementary Table S15). On the KEGG pathway database, the expanded gene families were functionally associated with the oil biosynthesis pathway, including fatty acid biosynthesis (ko00061), fatty acid elongation (ko00062), fatty acid degradation (ko00071), and glycerolipid metabolism (ko00561) (Supplementary Table S16). These findings suggest that *M. tetraphylla* displayed an enhanced ability for oil biosynthesis, a critical trait for flavor and quality in macadamia.

WGD (whole-genome duplication) events are of great importance in generating species diversity during evolution (Fu et al., 2021). We used 4-fold synonymous third-codon transversion (4DTv) to detect the WGD events in the *M. tetraphylla* genome. In a self-alignment of *M. tetraphylla*, a total of 581 syntenic genomic blocks covering 8,953 genes were identified in the macadamia genome. The orthology within macadamia genome showed 4DTv distance peaks at ∼0.10 and ∼0.42, respectively (Figure 2C), suggesting that two rounds of WGD events occurred in the macadamia genome. We also compared the *M. tetraphylla* genome with grape genome sequences, and a peak (4DTv ∼0.14) was observed. These results indicated that *M. tetraphylla* has undergo a species-specific WGD event after the divergence between *M. tetraphylla* and grape (Figure 2C), and *M. tetraphylla* shared an ancient WGD event (A*t*-γ) with grape (Figure 2C).

### Analysis of Oil Metabolism Genes

The high fat content of macadamia kernels is the most prominent feature of this fruit tree, especially the high content of unsaturated fatty acids, which determines its benefits to human health. According to the determination of our research team, macadamia nuts contain up to 80% fatty acids consisting of 13 different types. The three most abundant fatty acids are oleic acid (57–66%), palmitoleic acid (10–18%), and palmitic acid (10–18%), and the other ten fatty acids constitute <4% of the total fatty acids content**.**

**FIGURE 3:**
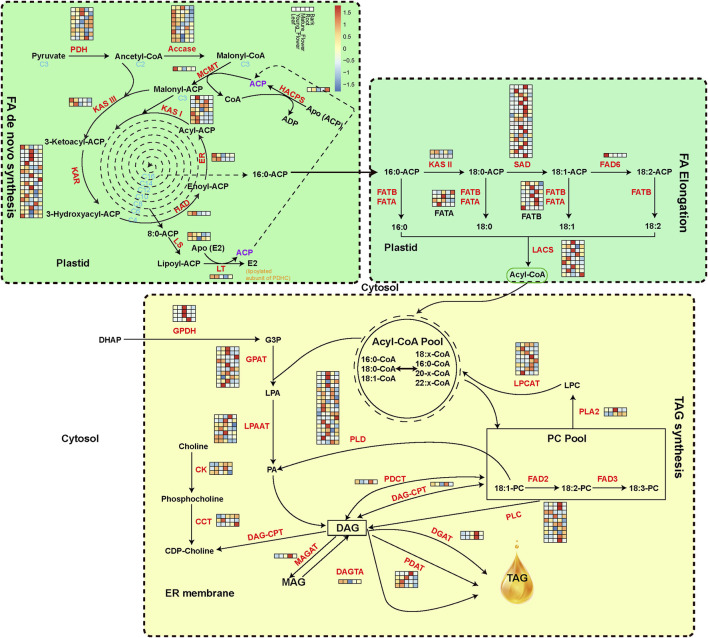
Expression level of oil biosynthesis-related genes. Acetyl-CoA is converted into C16 and C18 fatty acids in the plastid. TAG is synthesized in the endoplasmic reticulum and packed in the oil bodies. The isozymes and metabolites involved in oil biosynthesis were colored in red and black, respectively. The expression levels of oil-biosynthesis genes from leaf, young flower, mature flower, root and bark, are presented with the heat map.

The 187 genes encoding 33 crucial oil biosynthesis enzymes, including those involved in *de novo* fatty acid synthesis, elongation, and TAG assembly, were manually annotated to expound on the evolution of oil metabolism genes of *M. tetraphylla* (Figure 3). The RNA-seq data from leaves, young flowers, mature flowers, roots, and barks identified member genes from key enzyme gene families (Supplementary Table S17). The acetyl-CoA carboxylase (ACCase, EC: 6.4.1.2) is the key enzyme determining the metabolic pathways that lead to oil or protein biosynthesis in the seed (Chen et al., 1999). Ten ACCase genes were identified in the *M. tetraphylla* genome but exhibited diverse expression patterns in different tissues (Figure 3 and Supplementary Table S17). Gene *MTE004907* of the ACCase gene family was highly expressed in all tissues, but the expression levels were substantially higher in leaves and flowers than roots and barks.

The FAD protein family catalyzes the desaturation of fatty acids (Park et al., 2008). FAD2 and FAD3 are the main enzymes responsible for linoleic acid desaturation. FAD2 is reported to be accountable for polyunsaturated lipid synthesis in the developing seed of oil crops. Two *FAD2* genes were identified in the *M. tetraphylla* genome, but none was expressed in the five sequenced tissues. Other gene families, including *KAR*, *SAD*, and *PLD*, contained the maximum number of gene copies, implying their central role in the oil formation pathway.

### Genome-Wide Investigation of *WRKY* Gene Family

**FIGURE 4:**
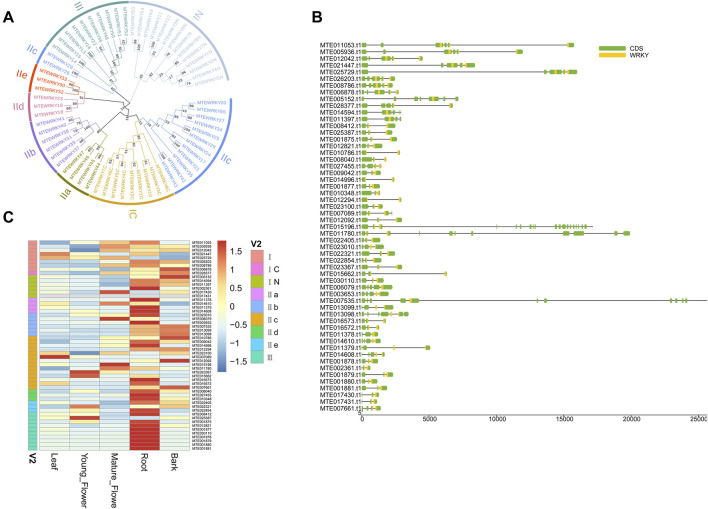
Genome-wide investigation of WRKY gene family. **(A)** Unrooted phylogenetic tree among WRKY domains from macadimia genome. **(B)** Exon-intron structure of *WRKY* genes. **(C)** Expression profiles of the *WRKY* genes.

The WRKY transcription factors (TFs) are among the most widespread gene families in higher plants (ülker and Somssich, 2004). The WRKY proteins play a crucial role in plant defense against biotic stress (Levée et al., 2009; Pandey and Somssich, 2009; Kloth et al., 2016). Fifty-five WRKY proteins were identified based on a WRKY domain and BLAST searches (Supplementary Table S18). The identified WRKY proteins were 134 aa (*MTE002361*) to 1,050 aa (*MTE011780*) long (Supplementary Table S18). Multiple sequence alignment was performed to check the phylogenetic relationship of the WRKY proteins. The WRKY domains covered three groups corresponding to the groups I, II, and III. Group II was dominant which contained 30 members (Figure 4A and Supplementary Table S18). As previously reported, the WRKY domains from the N-termini and C-termini were grouped into different clades, indicating that the two domains underwent parallel evolution (Tao et al., 2018). The *WRKY* genes possessed at least two exons, including the optimal number of exons to a maximum of twenty (Figure 4B). To further confirm whether different tissues influenced the expression level of *WRKY* genes, we calculated the FPKM (Fragments Per Kilobase per Million) value of each gene. Forty-one *WRKY* genes were expressed in all five sampled tissues, with 20 genes showing constitutive expression (FPKM >1 in all samples) (Supplementary Table S19). However, gene *MTE017431* was not expressed in any of the five sampled tissues, suggesting a pseudogene (Figure 4C). Interestingly, the *WRKY* genes showed higher transcript abundance in roots than other tissues (Figure 4C), suggesting that defense in macadamia primarily occurs in this organ, probably against soil microorganisms and pathogens.

## Discussion

*M. tetraphylla* L. Johnson, is a tropical to subtropical tree that has its origins in southeastern Queensland and northeastern New South Wales in Australia (Mulwa and Bhalla, 2000). It is highly valued for its versatile nut. However, production of macadamia is hampered by low yield. Here, we generated a chromosome-scale assembly of *M. tetraphylla* genome combing Oxford Nanopore Technologies and Hi-C technology. The N50 contig showed a striking 335.8-fold increase compared to *M. integrifolia*. Repeated sequence insertion has been proved to be a main force for the expansion of plant genome as observed in tea tree and rubber tree (Liu et al., 2020; Zhang et al., 2020). Similar to these species, *Gypsy*-type elements contributed the most to the macadamia tree genome.

We have identified lineage-specific genes that likely control the thick shells of macadamia, in particular genes encoding enzymes involved in the chitin catabolic process. Our comparative analyses indicate that *M. tetraphylla* is closely related to *N. nucifera* and diverged ∼132.2 million year ago. A high proportion of gene families related to fatty acid metabolism were expanded in the macadamia tree genome, indicating *M. tetraphylla* displayed an enhanced ability for oil biosynthesis. A similar result was observed in the tung tree, which is an economically important woody oil plant that produces tung oil rich in eleostearic acid (Zhang et al., 2019). A recent WGD event was also detected in the *M. tetraphylla* genome.

Like many other nuts, macadamia nuts are high in monounsaturated fatty acids. Monounsaturated fatty acids have been linked to reduced cholesterol in the blood (https://www.medicinalfoodnews.com/). A total of 187 genes encoding 33 crucial oil biosynthesis enzymes were identified. ACCase is a key enzyme determining the metabolic pathways toward oil or protein biosynthesis (Chen et al., 1999). Ten ACCase genes were identified but the expression levels varied, which may be a cause of different fatty acids content in different tissues. We have also observed the *KAR*, *SAD*, and *PLD* gene families contained the maximum number of gene copies, implying their central role in the oil formation pathway. A total of 55 *WRKY* genes were identified in the *M. tetraphylla* genome. We have found that most of the *WRKY* members showed higher expression level in roots, indicating that this tissue may play a crucial role in plant defense against soil microorganisms and pathogens. We believe that the genome sequencing efforts summarized in this study would facilitate the breeding of this elite nut tree.

## Conclusion

This paper presents the sequencing, assembly, and annotation of the *M. tetraphylla* genome. The extensive datasets and analyses presented will provide novel insights into the genome evolution of this species and facilitate the breeding strategies for genetic improvement. As a woody plant, breeding for any new variety often takes decades, and molecular marker-assisted screening offers a practical approach to shorten the breeding cycle. The genomic data obtained in this study will also provide the primary data for mining genes and developing molecular markers hence the foundation for molecular breeding of macadamia.

## Data Availability

The datasets presented in this study can be found in online repositories. The names of the repository/repositories and accession number(s) can be found in the article/Supplementary Material.

## References

[B1] Ahmad Termizi A. Hardner C. Batley J. Nock C. J. Hayashi S. Montenegro J. (2014). SNP Analysis of Macadamia Integrifolia Chloroplast Genomes to Determine the Genetic Structure of Wild Populations. XXIX Int. Hortic. Congress Hortic. Sustaining Lives 1109, 175–180. 10.17660/actahortic.2016.1109.29

[B2] Akinsanmi O. A. Neal J. Drenth A. Topp B. (2017). Characterization of Accessions and Species ofMacadamiato Stem Infection byPhytophthora Cinnamomi. Plant Pathol. 66, 186–193. 10.1111/ppa.12566

[B3] Bairoch A. Apweiler R. (2000). The SWISS-PROT Protein Sequence Database and its Supplement TrEMBL in 2000. Nucleic Acids Res. 28, 45–48. 10.1093/nar/28.1.45 10592178 PMC102476

[B4] Bao W. Kojima K. K. Kohany O. (2015). Repbase Update, a Database of Repetitive Elements in Eukaryotic Genomes. Mobile Dna 6, 11. 10.1186/s13100-015-0041-9 26045719 PMC4455052

[B5] Benson G. (1999). Tandem Repeats Finder: a Program to Analyze DNA Sequences. Nucleic Acids Res. 27, 573–580. 10.1093/nar/27.2.573 9862982 PMC148217

[B6] Birney E. Clamp M. Durbin R. (2004). GeneWise and Genomewise. Genome Res. 14, 988–995. 10.1101/gr.1865504 15123596 PMC479130

[B7] Chagné D. (2015). “Whole Genome Sequencing of Fruit Tree Species,” in Advances in Botanical Research. Editors Plomion C. Adam-Blondon A. F. (Academic Press), 1–37. 10.1016/bs.abr.2015.04.004

[B8] Chen J. Q. Lang C. X. Hu Z. H. Liu Z. H. Huang R. Z. (1999). Antisense PEP Gene Regulates to Ratio of Protein and Lipid Content in *Brassica Napus* Seeds. J. Agric. Biotechnol. 7, 316–320. 10.3969/j.issn.1674-7968.1999.04.003

[B9] Daehwan K. Ben L. Salzberg S. L. (2015). HISAT: a Fast Spliced Aligner with Low Memory Requirements. Nat. Methods 12, 357–360. 10.1038/nmeth.3317 25751142 PMC4655817

[B10] De Bie T. Cristianini N. Demuth J. P. Hahn M. W. (2006). CAFE: a Computational Tool for the Study of Gene Family Evolution. Bioinformatics 22, 1269–1271. 10.1093/bioinformatics/btl097 16543274

[B11] Doležel J. Greilhuber J. Suda J. (2007). Estimation of Nuclear DNA Content in Plants Using Flow Cytometry. Nat. Protoc. 2, 2233–2244. 10.1038/nprot.2007.310 17853881

[B12] Dudchenko O. Batra S. S. Omer A. D. Nyquist S. K. Hoeger M. Durand N. C. (2017). De Novo assembly of the *Aedes aegypti* Genome Using Hi-C Yields Chromosome-Length Scaffolds. Science 356, 92–95. 10.1126/science.aal3327 28336562 PMC5635820

[B13] Edgar R. C. (2004). MUSCLE: a Multiple Sequence Alignment Method with Reduced Time and Space Complexity. BMC Bioinformatics 5, 113. 10.1186/1471-2105-5-113 15318951 PMC517706

[B14] Edgar R. C. Myers E. W. (2005). PILER: Identification and Classification of Genomic Repeats. Bioinformatics 21, i152–i158. 10.1093/bioinformatics/bti1003 15961452

[B15] Fu A. Wang Q. Mu J. Ma L. Wen C. Zhao X. (2021). Combined Genomic, Transcriptomic, and Metabolomic Analyses Provide Insights into Chayote (*Sechium Edule*) Evolution and Fruit Development. Hortic. Res. 8, 35. 10.1038/s41438-021-00487-1 33517348 PMC7847470

[B16] Garg M. L. Blake R. J. Wills R. B. H. Clayton E. H. (2007). Macadamia Nut Consumption Modulates Favourably Risk Factors for Coronary Artery Disease in Hypercholesterolemic Subjects. Lipids 42, 583–587. 10.1007/s11745-007-3042-8 17437143

[B17] Garg M. L. Blake R. J. Wills R. B. H. (2003). Macadamia Nut Consumption Lowers Plasma Total and LDL Cholesterol Levels in Hypercholesterolemic Men. J. Nutr. 133, 1060–1063. 10.1093/jn/133.4.1060 12672919

[B18] Griffiths-Jones S. Moxon S. Marshall M. Khanna A. Eddy S. R. Bateman A. (2005). Rfam: Annotating Non-coding RNAs in Complete Genomes. Nucleic Acids Res. 33, D121–D124. 10.1093/nar/gki081 15608160 PMC540035

[B19] Haas B. J. Delcher A. L. Mount S. M. Wortman J. R. Smith R. K. Jr Hannick L. I. (2003). Improving the Arabidopsis Genome Annotation Using Maximal Transcript Alignment Assemblies. Nucleic Acids Res. 31, 5654–5666. 10.1093/nar/gkg770 14500829 PMC206470

[B20] Haas B. J. Salzberg S. L. Zhu W. Pertea M. Allen J. E. Orvis J. (2008). Automated Eukaryotic Gene Structure Annotation Using EVidenceModeler and the Program to Assemble Spliced Alignments. Genome Biol. 9, R7. 10.1186/gb-2008-9-1-r7 18190707 PMC2395244

[B21] Hardner C. (2016). Macadamia Domestication in Hawai'i. Genet. Resour. Crop Evol. 63, 1411–1430. 10.1007/s10722-015-0328-1

[B22] Hibrand Saint-Oyant L. Ruttink T. Hamama L. Kirov I. Lakhwani D. Zhou N. N. (2018). A High-Quality Genome Sequence of *Rosa Chinensis* to Elucidate Ornamental Traits. Nat. Plants 4, 473–484. 10.1038/s41477-018-0166-1 29892093 PMC6786968

[B23] Initiative A. G. (2000). Analysis of the Genome Sequence of the Flowering Plant *Arabidopsis thaliana* . Nature 408, 796–815. 10.1038/35048692 11130711

[B24] Jones P. Binns D. Chang H.-Y. Fraser M. Li W. Mcanulla C. (2014). InterProScan 5: Genome-Scale Protein Function Classification. Bioinformatics 30, 1236–1240. 10.1093/bioinformatics/btu031 24451626 PMC3998142

[B25] Kaijser A. Dutta P. Savage G. (2000). Oxidative Stability and Lipid Composition of Macadamia Nuts Grown in New Zealand. Food Chem. 71, 67–70. 10.1016/s0308-8146(00)00132-1

[B26] Kim D. Langmead B. Salzberg S. L. (2015). HISAT: a Fast Spliced Aligner with Low Memory Requirements. Nat. Methods 12, 357–360. 10.1038/nmeth.3317 25751142 PMC4655817

[B27] Kloth K. J. Wiegers G. L. Busscher-Lange J. Van Haarst J. C. Kruijer W. Bouwmeester H. J. (2016). AtWRKY22 Promotes Susceptibility to Aphids and Modulates Salicylic Acid and Jasmonic Acid Signalling. Exbotj 67, 3383–3396. 10.1093/jxb/erw159 PMC4892728 27107291

[B28] Koren S. Walenz B. P. Berlin K. Miller J. R. Bergman N. H. Phillippy A. M. (2017). Canu: Scalable and Accurate Long-Read Assembly via Adaptive K-Mer Weighting and Repeat Separation. Genome Res. 27, 722–736. 10.1101/gr.215087.116 28298431 PMC5411767

[B29] Korf I. (2004). Gene Finding in Novel Genomes. BMC Bioinformatics 5, 59. 10.1186/1471-2105-5-59 15144565 PMC421630

[B30] Lagesen K. Hallin P. Rødland E. A. Stærfeldt H.-H. Rognes T. Ussery D. W. (2007). RNAmmer: Consistent and Rapid Annotation of Ribosomal RNA Genes. Nucleic Acids Res. 35, 3100–3108. 10.1093/nar/gkm160 17452365 PMC1888812

[B31] Langmead B. Salzberg S. L. (2012). Fast Gapped-Read Alignment with Bowtie 2. Nat. Methods 9, 357–359. 10.1038/nmeth.1923 22388286 PMC3322381

[B32] Levée V. Major I. Levasseur C. Tremblay L. Mackay J. Séguin A. (2009). Expression Profiling and Functional Analysis of *Populus* WRKY23 Reveals a Regulatory Role in Defense. New Phytol. 184, 48–70. 10.1111/j.1469-8137.2009.02955.x 19674332

[B33] Li H. (2018). Minimap2: Pairwise Alignment for Nucleotide Sequences. Bioinformatics 34, 3094–3100. 10.1093/bioinformatics/bty191 29750242 PMC6137996

[B34] Li L. Stoeckert C. J. Roos D. S. (2003). OrthoMCL: Identification of Ortholog Groups for Eukaryotic Genomes. Genome Res. 13, 2178–2189. 10.1101/gr.1224503 12952885 PMC403725

[B35] Liu J. Niu Y.-F. Ni S.-B. He X.-Y. Shi C. (2017). Complete Chloroplast Genome of a Subtropical Fruit Tree *Macadamia Ternifolia* (Proteaceae). Mitochondrial DNA B 2, 738–739. 10.1080/23802359.2017.1390401 PMC7800794 33473964

[B36] Liu J. Niu Y.-F. Ni S.-B. He X.-Y. Zheng C. Liu Z.-Y. (2018). The Whole Chloroplast Genome Sequence of *Macadamia Tetraphylla* (Proteaceae). Mitochondrial DNA Part B 3, 1276–1277. 10.1080/23802359.2018.1532836 33474491 PMC7799527

[B37] Liu J. Shi C. Shi C.-C. Li W. Zhang Q.-J. Zhang Y. (2020). The Chromosome-Based Rubber Tree Genome Provides New Insights into Spurge Genome Evolution and Rubber Biosynthesis. Mol. Plant 13, 336–350. 10.1016/j.molp.2019.10.017 31838037

[B38] Lowe T. M. Eddy S. R. (1999). A Computational Screen for Methylation Guide snoRNAs in Yeast. Science 283, 1168–1171. 10.1126/science.283.5405.1168 10024243

[B39] Mario S. Rasmus S. Stephan W. Burkhard M. (2004). AUGUSTUS: a Web Server for Gene Finding in Eukaryotes. Nucleic Acids Res. 32, 309–312. 10.1093/nar/gkh379 PMC441517 15215400

[B40] Marçais G. Kingsford C. (2011). A Fast, Lock-free Approach for Efficient Parallel Counting of Occurrences of K-Mers. Bioinformatics 27, 764–770. 10.1093/bioinformatics/btr011 21217122 PMC3051319

[B41] Ming R. Vanburen R. Liu Y. Yang M. Han Y. Li L. T. (2013). Genome of the Long-Living Sacred lotus (*Nelumbo nucifera* Gaertn.). Genome Biol. 14, R41. 10.1186/gb-2013-14-5-r41 23663246 PMC4053705

[B42] Mulwa R. Bhalla P. (2000). *In Vitro* shoot Multiplication ofMacadamia tetraphyllaL. Johnson. J. Hortic. Sci. Biotechnol. 75, 1–5. 10.1080/14620316.2000.11511192

[B43] Navarro S. L. B. Rodrigues C. E. C. (2018). Macadamia Oil Extraction with Alcoholic Solvents: Yield and Composition of Macadamia Oil and Production of Protein Concentrates from Defatted Meal. Eur. J. Lipid Sci. Technol. 120, 1800092. 10.1002/ejlt.201800092

[B44] Nawrocki E. P. Kolbe D. L. Eddy S. R. (2009). Infernal 1.0: Inference of RNA Alignments. Bioinformatics 25, 1335–1337. 10.1093/bioinformatics/btp157 19307242 PMC2732312

[B45] Neal J. M. Hardner C. M. Gross C. L. (2010). Population Demography and Fecundity Do Not Decline with Habitat Fragmentation in the Rainforest Tree *Macadamia Integrifolia* (Proteaceae). Biol. Conservation 143, 2591–2600. 10.1016/j.biocon.2010.06.029

[B46] Nock C. J. Baten A. Barkla B. J. Furtado A. Henry R. J. King G. J. (2016). Genome and Transcriptome Sequencing Characterises the Gene Space of *Macadamia Integrifolia* (Proteaceae). BMC Genomics 17, 937. 10.1186/s12864-016-3272-3 27855648 PMC5114810

[B47] Nock C. J. Baten A. King G. J. (2014). Complete Chloroplast Genome of Macadamia Integrifoliaconfirms the Position of the Gondwanan Early-Diverging Eudicot Family Proteaceae. BMC Genomics 15, S13. 10.1186/1471-2164-15-s9-s13 PMC4290595 25522147

[B48] Pandey S. P. Somssich I. E. (2009). The Role of WRKY Transcription Factors in Plant Immunity. Plant Physiol. 150, 1648–1655. 10.1104/pp.109.138990 19420325 PMC2719123

[B49] Park J.-Y. Kim D.-K. Wang Z.-M. Lu P. Park S.-C. Lee J.-S. (2008). Production and Characterization of Biodiesel from Tung Oil. Appl. Biochem. Biotechnol. 148, 109–117. 10.1007/s12010-007-8082-2 18418744

[B50] Pertea M. Kim D. Pertea G. M. Leek J. T. Salzberg S. L. (2016). Transcript-level Expression Analysis of RNA-Seq Experiments with HISAT, StringTie and Ballgown. Nat. Protoc. 11, 1650–1667. 10.1038/nprot.2016.095 27560171 PMC5032908

[B51] Pisanu P. C. Gross C. L. Flood L. (2009). Reproduction in Wild Populations of the Threatened TreeMacadamia Tetraphylla: Interpopulation Pollen Enriches Fecundity in a Declining Species. Biotropica 41, 391–398. 10.1111/j.1744-7429.2008.00484.x

[B52] Price A. L. Jones N. C. Pevzner P. A. (2005). *De Novo* identification of Repeat Families in Large Genomes. Bioinformatics 21, i351–i358. 10.1093/bioinformatics/bti1018 15961478

[B53] Ruan J. Li H. (2020). Fast and Accurate Long-Read Assembly with Wtdbg2. Nat. Methods. 17 (2), 155–158. 10.1038/s41592-019-0669-3 31819265 PMC7004874

[B54] Schattner P. Brooks A. N. Lowe T. M. (2005). The tRNAscan-SE, Snoscan and snoGPS Web Servers for the Detection of tRNAs and snoRNAs. Nucleic Acids Res. 33, W686–W689. 10.1093/nar/gki366 15980563 PMC1160127

[B55] Shapcott A. Powell M. (2011). Demographic Structure, Genetic Diversity and Habitat Distribution of the Endangered, Australian Rainforest Tree *Macadamia Jansenii* Help Facilitate an Introduction Program. Aust. J. Bot. 59, 215–225. 10.1071/bt10132

[B56] She R. Chu J. S.-C. Wang K. Pei J. Chen N. (2009). GenBlastA: Enabling BLAST to Identify Homologous Gene Sequences. Genome Res. 19, 143–149. 10.1101/gr.082081.108 18838612 PMC2612959

[B57] Simão F. A. Waterhouse R. M. Ioannidis P. Kriventseva E. V. Zdobnov E. M. (2015). BUSCO: Assessing Genome Assembly and Annotation Completeness with Single-Copy Orthologs. Bioinformatics 31, 3210–3212. 10.1093/bioinformatics/btv351 26059717

[B58] Stamatakis A. (2014). RAxML Version 8: a Tool for Phylogenetic Analysis and post-analysis of Large Phylogenies. Bioinformatics 30, 1312–1313. 10.1093/bioinformatics/btu033 24451623 PMC3998144

[B59] Tao X. Chengjie C. Chuhao L. Jiarou L. Chaoyang L. Yehua H. (2018). Genome-wide Investigation of *WRKY* Gene Family in Pineapple: Evolution and Expression Profiles during Development and Stress. BMC Genomics 19, 490. 10.1186/s12864-018-4880-x 29940851 PMC6019807

[B60] Tarailo‐Graovac M. Chen N. (2009). Using RepeatMasker to Identify Repetitive Elements in Genomic Sequences. Curr. Protoc. Bioinformatics 25, 4. 10.1002/0471250953.bi0410s25 19274634

[B61] Thiel T. Michalek W. Varshney R. Graner A. (2003). Exploiting EST Databases for the Development and Characterization of Gene-Derived SSR-Markers in Barley (*Hordeum Vulgare* L.). Theor. Appl. Genet. 106, 411–422. 10.1007/s00122-002-1031-0 12589540

[B62] Toft B. D. Alam M. Topp B. (2018). Estimating Genetic Parameters of Architectural and Reproductive Traits in Young Macadamia Cultivars. Tree Genet. Genomes 14, 50. 10.1007/s11295-018-1265-x

[B63] Trueman S. Turnbull C. (1994). Effects of Cross-Pollination and Flower Removal on Fruit Set in Macadamia. Ann. Bot 73, 23–32. 10.1006/anbo.1994.1003

[B64] Ülker B. Somssich I. E. (2004). WRKY Transcription Factors: from DNA Binding towards Biological Function. Curr. Opin. Plant Biol. 7, 491–498. 10.1016/j.pbi.2004.07.012 15337090

[B65] Velasco R. Zharkikh A. Affourtit J. Dhingra A. Cestaro A. Kalyanaraman A. (2010). The Genome of the Domesticated Apple (Malus × Domestica Borkh.). Nat. Genet. 42, 833–839. 10.1038/ng.654 20802477

[B66] Vurture G. W. Sedlazeck F. J. Nattestad M. Underwood C. J. Fang H. Gurtowski J. (2017). GenomeScope: Fast Reference-free Genome Profiling from Short Reads. Bioinformatics 33, 2202–2204. 10.1093/bioinformatics/btx153 28369201 PMC5870704

[B67] Walker B. J. Abeel T. Shea T. Priest M. Abouelliel A. Sakthikumar S. (2014). Pilon: an Integrated Tool for Comprehensive Microbial Variant Detection and Genome Assembly Improvement. PLoS One 9, e112963. 10.1371/journal.pone.0112963 25409509 PMC4237348

[B68] Wang Y. Tang H. Debarry J. D. Tan X. Li J. Wang X. (2012). MCScanX: a Toolkit for Detection and Evolutionary Analysis of Gene Synteny and Collinearity. Nucleic Acids Res. 40, e49. 10.1093/nar/gkr1293 22217600 PMC3326336

[B69] Yuki M. Masumi I. Shujiro O. Yoshizawa A. C. Minoru K. (2007). KAAS: an Automatic Genome Annotation and Pathway Reconstruction Server. Nucleic Acids Res. 35, 182–185. 10.1093/nar/gkm321 PMC1933193 17526522

[B70] Zhang L. Li X. Ma B. Gao Q. Du H. Han Y. (2017). The Tartary Buckwheat Genome Provides Insights into Rutin Biosynthesis and Abiotic Stress Tolerance. Mol. Plant 10, 1224–1237. 10.1016/j.molp.2017.08.013 28866080

[B71] Zhang L. Liu M. Long H. Dong W. Pasha A. Esteban E. (2019). Tung Tree (*Vernicia Fordii*) Genome Provides a Resource for Understanding Genome Evolution and Improved Oil Production. Genomics, Proteomics & Bioinformatics 17, 558–575. 10.1016/j.gpb.2019.03.006 PMC7212303 32224189

[B72] Zhang Q.-J. Li W. Li K. Nan H. Shi C. Zhang Y. (2020). The Chromosome-Level Reference Genome of tea Tree Unveils Recent Bursts of Non-autonomous LTR Retrotransposons in Driving Genome Size Evolution. Mol. Plant 13, 935–938. 10.1016/j.molp.2020.04.009 32353626

