# Supplementary material for: The Chromosome-Scale Reference Genome of Macadamia tetraphylla Provides Insights Into Fatty Acid Biosynthesis
Source: Front Genet. 2022 Feb 23;13:835363. doi: 10.3389/fgene.2022.835363 (PMC8906886; doi:10.3389/fgene.2022.835363)
Supplement: Supplementary file 1 [file DataSheet3.docx]

**Tables**

**Supplemental** **Table 2. Statistics of Oxford Nanopore long-reads used for genome assembly.**

| **Pass Reads Number** | **Pass Reads Bases (bp)** | **Mean Reads Length (bp)** | **Reads N50 Length (bp)** | **Mean Read Quality** |
| --- | --- | --- | --- | --- |
| 3,382,098 | 68,173,294,275 | 20,157 | 24,256 | 7.6 |

**Supplemental** **Table 3. Statistics of Illumina sequencing reads used for genome and transcriptome assembly.**

| **Data Type** | **Library** | **Insert Size (bp)** | **Read Length (bp)** | **Total Bases (Gb)** |
| --- | --- | --- | --- | --- |
| DNA | PE01 | 500 | 150 | 88.27 |
| RNA | T01 | 250 | 150 | 6.49 |
|  | T02 | 250 | 150 | 6.58 |
|  | T03 | 250 | 150 | 8.55 |
|  | T04 | 250 | 150 | 6.40 |
|  | T05 | 250 | 150 | 6.28 |

**Supplemental** **Table 4. Comparison between genome assemblies for two macadamia species.**

| **Genome features** | ***M. tetraphylla*** | ***M. integrifolia*** |
| --- | --- | --- |
| Estimated genome size (Mb) | 758 | 652 |
| Total length of assembly (bp) | 750,866,652 | 518,489,877 |
| No. of contigs | 4,335 | 210,726 |
| Contig N50 (bp) | 1,182,547 | 3,522 |
| Longest contig (bp) | 9,780,764 | 379,349 |
| No. of scaffolds | 1,059 | 193,493 |
| Scaffold N50 (bp) | 51,109,939 | 4,745 |
| Longest scaffold (bp) | 87,095,162 | 643,490 |

**Supplementary Table 5. Statistic of Hi-C sequencing data for *M. tetraphylla* genome.**

| **Sample** | **Read Number** | **Base Number** | **Q30 Rate (%)** |
| --- | --- | --- | --- |
| 1 | 256,965,245 | 76,782,857,040‬‬‬‬ | 92.98 |
| 2 | 276,399,207 | 82,588,376,466‬‬‬‬ | 93.85 |
| Total | 533,364,452 | 159,371,233,506 |  |

**Supplementary Table 6. Chromosome lengths of the assembled *M. tetraphylla* genome.**

| **Chromosome ID** | **Length (bp)** | **GC Content (%)** |
| --- | --- | --- |
| Chr1 | 87,095,162 | 39.48 |
| Chr2 | 39,037,887 | 38.60 |
| Chr3 | 55,171,544 | 39.14 |
| Chr4 | 55,071,846 | 38.50 |
| Chr5 | 63,246,543 | 38.96 |
| Chr6 | 61,838,353 | 40.56 |
| Chr7 | 45,693,739 | 38.76 |
| Chr8 | 51,968,923 | 39.14 |
| Chr9 | 50,845,093 | 39.17 |
| Chr10 | 46,644,038 | 39.01 |
| Chr11 | 51,109,939 | 38.90 |
| Chr12 | 40,541,444 | 40.10 |
| Chr13 | 35,980,175 | 39.71 |
| Chr14 | 30,928,968 | 38.77 |

**Supplemental** **Table 7. Evaluation of genome assembly using different methods.**

| **Method** | **Feature** | **Value** |
| --- | --- | --- |
| **Illumina reads mapping** | **Reads number** | 573,843,844 |
|  | **Mapped number** | 540,860,068 |
|  | **Mapping rate (%)** | 94.25 |
|  | **Properly mapped rate (%)** | 87.84 |
| **ONT reads mapping** | **Reads number** | 12,449,453 |
|  | **Mapped number** | 12,426,184 |
|  | **Mapping rate (%)** | 99.81 |
| **BUSCO** | **Complete number** | 1250 |
|  | **Fragmented number** | 42 |
|  | **Missing rate (%)** | 10.3 |
| **RNA-Seq** | **Reads number** | 146,233,111 |
|  | **Mapped number** | 134,531,219 |
|  | **Mapping rate (%)** | 92.00 |

**Supplemental** **Table 8. Annotation of repeat sequences in the *M. tetraphylla* genome.**

| **Repeat elements** | | | | **Copy number** | **Length (bp)** | **Percentage (%)** |
| --- | --- | --- | --- | --- | --- | --- |
| Retrotransposons | | | | 214,430 | 262,347,793 | 34.95 |
|  | Non-LTR retrotransposons | |  | 72,628 | 44,629,376 | 5.95 |
|  | LTR retrotransposons | |  | 141,802 | 217,718,417 | 29.01 |
|  |  | *Gypsy* | | 88,052 | 165,151,480 | 22.00 |
|  |  | *Copia* | | 46,649 | 44,586,669 | 5.94 |
|  |  | Other | | 7,101 | 7,980,268 | 1.06 |
| DNA transposons | | | | 11,167 | 7,521,183 | 1.00 |
| Unclassified elements | | | | 947,252 | 191,137,916 | 25.47 |
| Total | | | | 1,172,849 | 461,006,892 | 61.42 |

**Supplemental** **Table 9. Summary of simple sequence repeats (SSRs) identified in the *M. tetraphylla* genome.**

| **Type** | **Unit size** | **Number** | **Length (bp)** |
| --- | --- | --- | --- |
| Monomer | n >= 12 | 171,372 | 2,565,684 |
| Dimer | n >= 6 | 113,278 | 2,454,290 |
| Trimer | n >= 4 | 87,467 | 1,412,550 |
| Tetramer | n >= 3 | 81,444 | 1,079,288 |
| Pentamer | n >= 3 | 37,088 | 617,310 |
| Hexamer | n >= 3 | 20,244 | 456,330 |
| Total Number |  | 510,893 | 8,585,452 |

**Supplemental** **Table 10. Prediction of protein-coding genes identified in the *M. tetraphylla* genome.**

| **Gene features** |  |
| --- | --- |
| Total number of protein-coding genes | 31,571 |
| Gene length in genome (%) | 25.47 |
| Average gene length (bp) | 6,055 |
| Average exon length (bp) | 222 |
| Average CDS length (bp) | 1,123 |
| Average intron length (bp) | 1,213 |
| Average exons per gene | 5.1 |
| Gene density (gene per Mb) | 42.04 |

**Supplemental** **Table 11. Functional annotation of the protein-coding genes.**

| **Database** | **Number** | **Percentage (%)** |
| --- | --- | --- |
| **Swiss-Prot** | 22,869 | 72.44 |
| **KEGG** | 8,303 | 26.30 |
| **InterPro** | 29,052 | 92.02 |
| **GO** | 17,864 | 56.58 |
| **Pfam** | 21,925 | 69.45 |
| **Total** | 29,233 | 92.59 |

**Supplemental** **Table 12. Annotation of non-coding RNA genes identified in the *M. tetraphylla* genome.**

| **Type** | **Number** | **Average length (bp)** | **Total length (bp)** | **Perceentage (%)** |
| --- | --- | --- | --- | --- |
| tRNA | 1,286 | 74.34 | 95,604 | 0.0127 |
| rRNA (8s) | 210 | 112.94 | 23,718 | 0.0032 |
| rRNA (18s) | 177 | 1,886.61 | 333,931 | 0.0445 |
| rRNA (28s) | 155 | 4,333.59 | 671,707 | 0.0895 |
| snRNA | 251 | 122.01 | 30,624 | 0.0041 |
| snoRNA | 74 | 114.12 | 8,445 | 0.0011 |

**Supplemental Table 13.** **Comparisons of genes and gene families among plant species investigated.**

| **Species** | **Family number** | **Genes in families** | **Unique families** | **Unique genes** | **Average genes per family** |
| --- | --- | --- | --- | --- | --- |
| Arabidopsis | 12,651 | 23,470 | 815 | 2,939 | 1.86 |
| Macadamia | 12,250 | 23,749 | 965 | 4,337 | 1.94 |
| Lotus | 12,001 | 20,078 | 446 | 1,413 | 1.67 |
| Coffee | 12,917 | 20,624 | 600 | 1,864 | 1.60 |
| Rice | 12,425 | 25,532 | 1,978 | 7,570 | 2.05 |
| Rose | 15,319 | 31,819 | 2,247 | 7,835 | 2.08 |
| Kiwifruit | 12,624 | 26,249 | 1,268 | 3,409 | 2.08 |
| Populus | 14,240 | 33,427 | 2,247 | 7,835 | 2.35 |
| CORE | 6,823 | 107,264 |  |  |  |
| TOTAL | 24,346 | 204,948 |  |  |  |
