# Supplementary material for: The Chromosome-Scale Reference Genome of Macadamia tetraphylla Provides Insights Into Fatty Acid Biosynthesis
Source: Front Genet. 2022 Feb 23;13:835363. doi: 10.3389/fgene.2022.835363 (PMC8906886; doi:10.3389/fgene.2022.835363)
Supplement: Supplementary file 2 [file DataSheet1.docx]

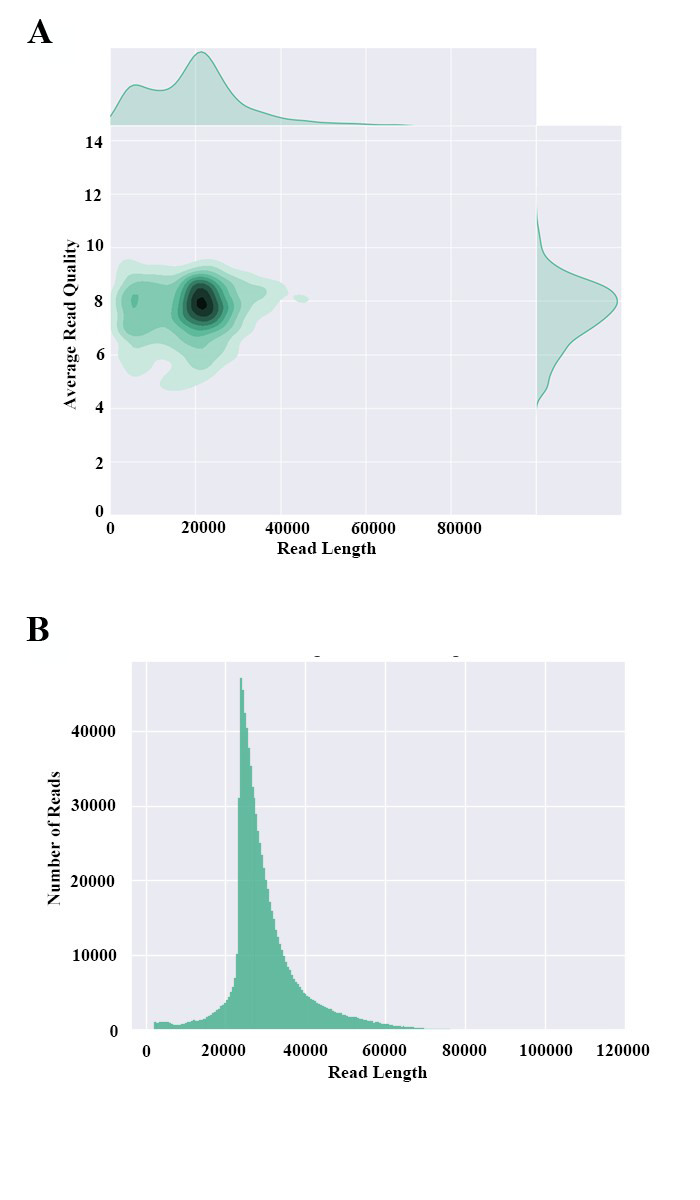


**Supplemental** **Figure 1. Length distribution of Oxford Nanopore long reads. (A)** Read length and read quality of the original reads; **(B)** Read length of the corrected reads.

**
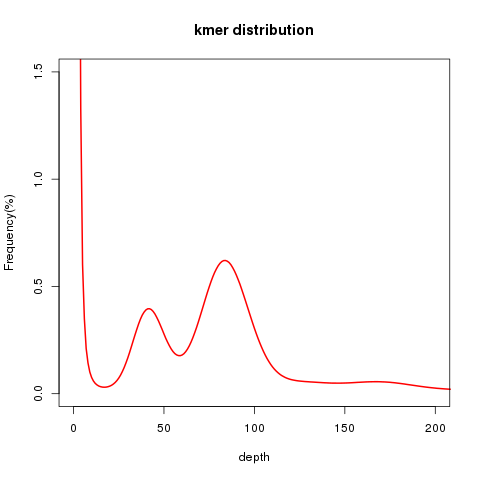
**

**Supplemental** **Figure 2. The 17-mer distribution of Illumina sequencing reads from the *M. tetraphylla* genome.**

**
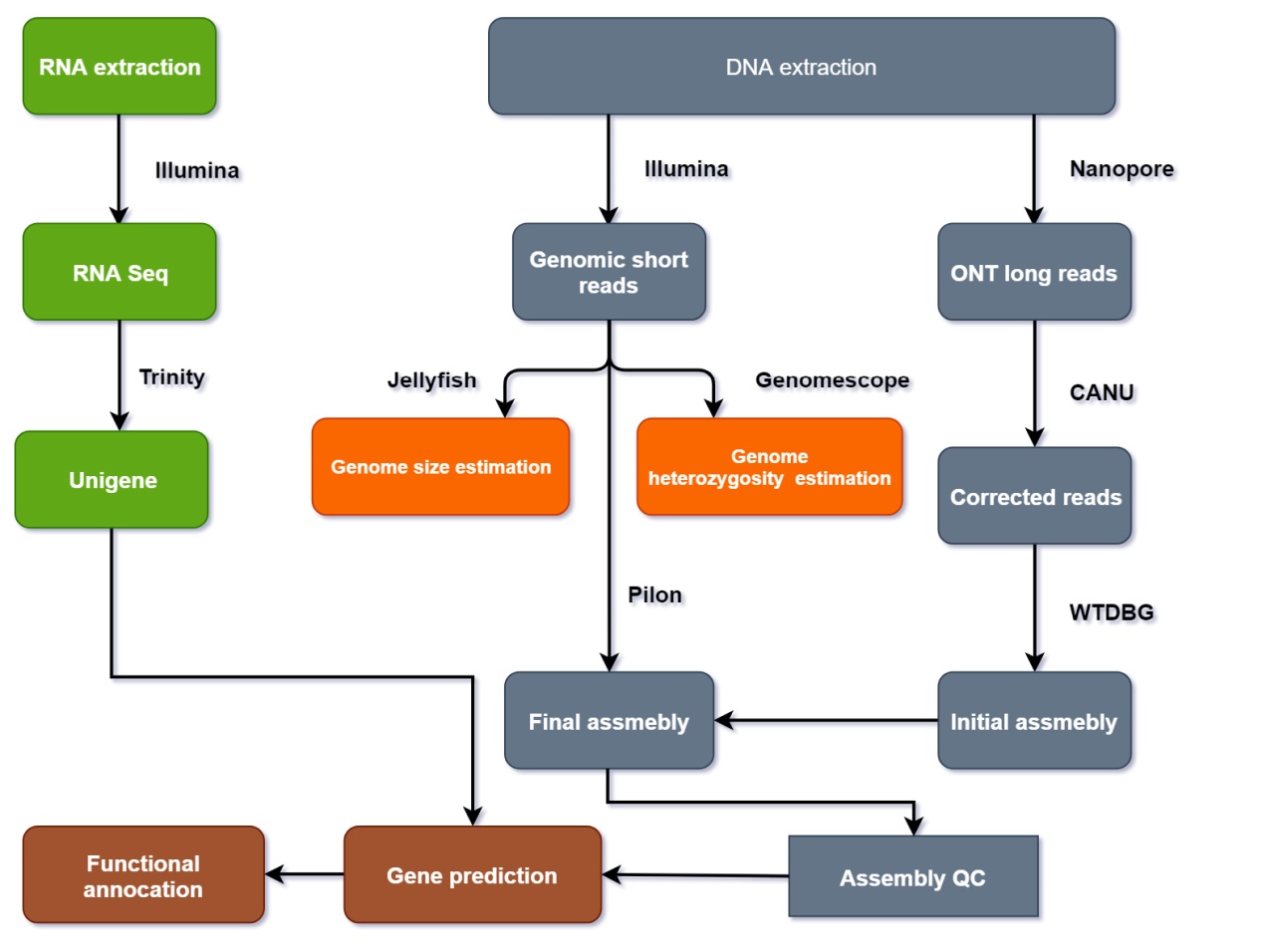
**

**Supplemental** **Figure 3. Overview of the pipeline used in this study.**

**
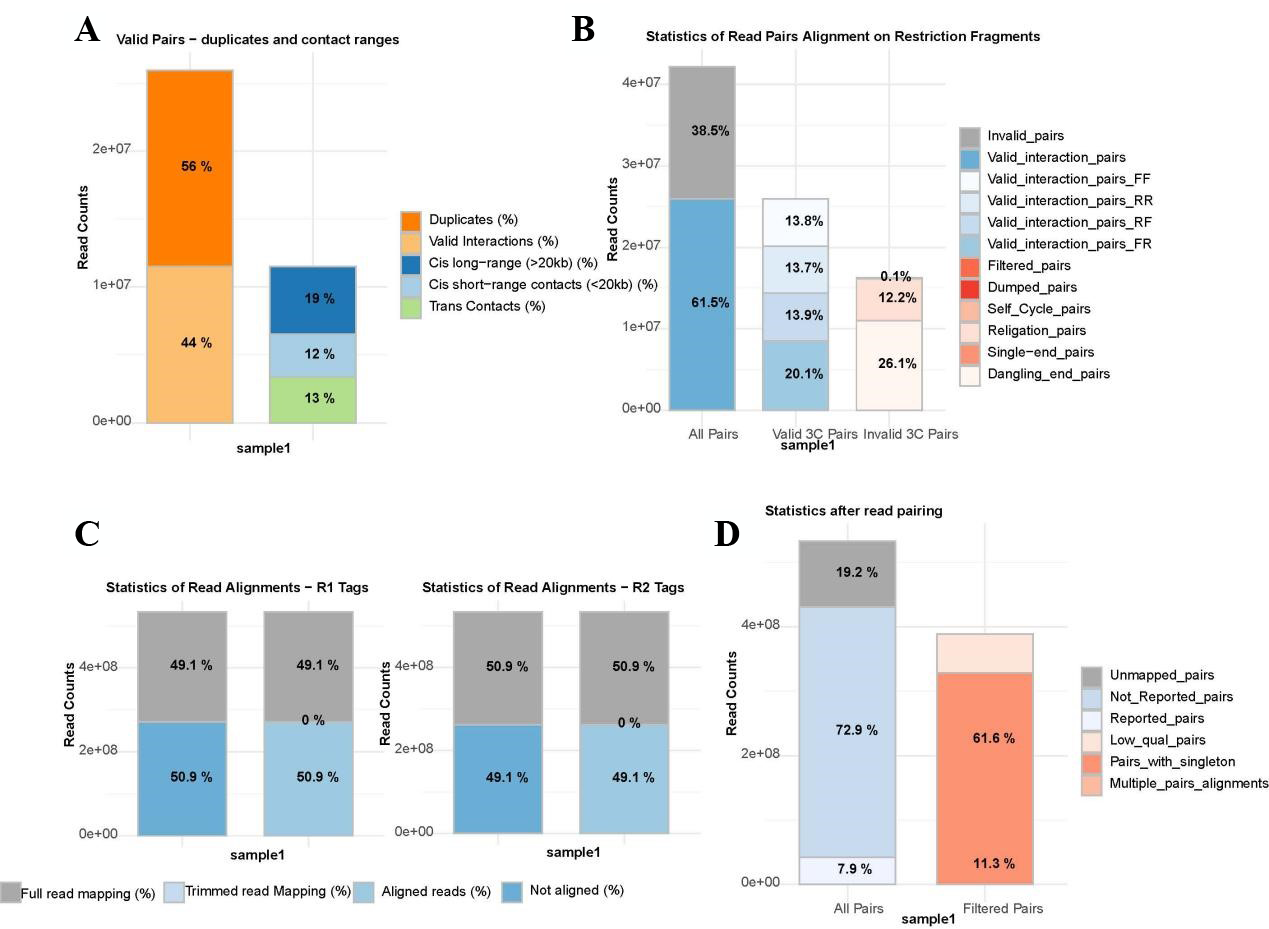
**

**Supplemental** **Figure 4. Statics of Hi-C reads mapping for genome assembly.**

**
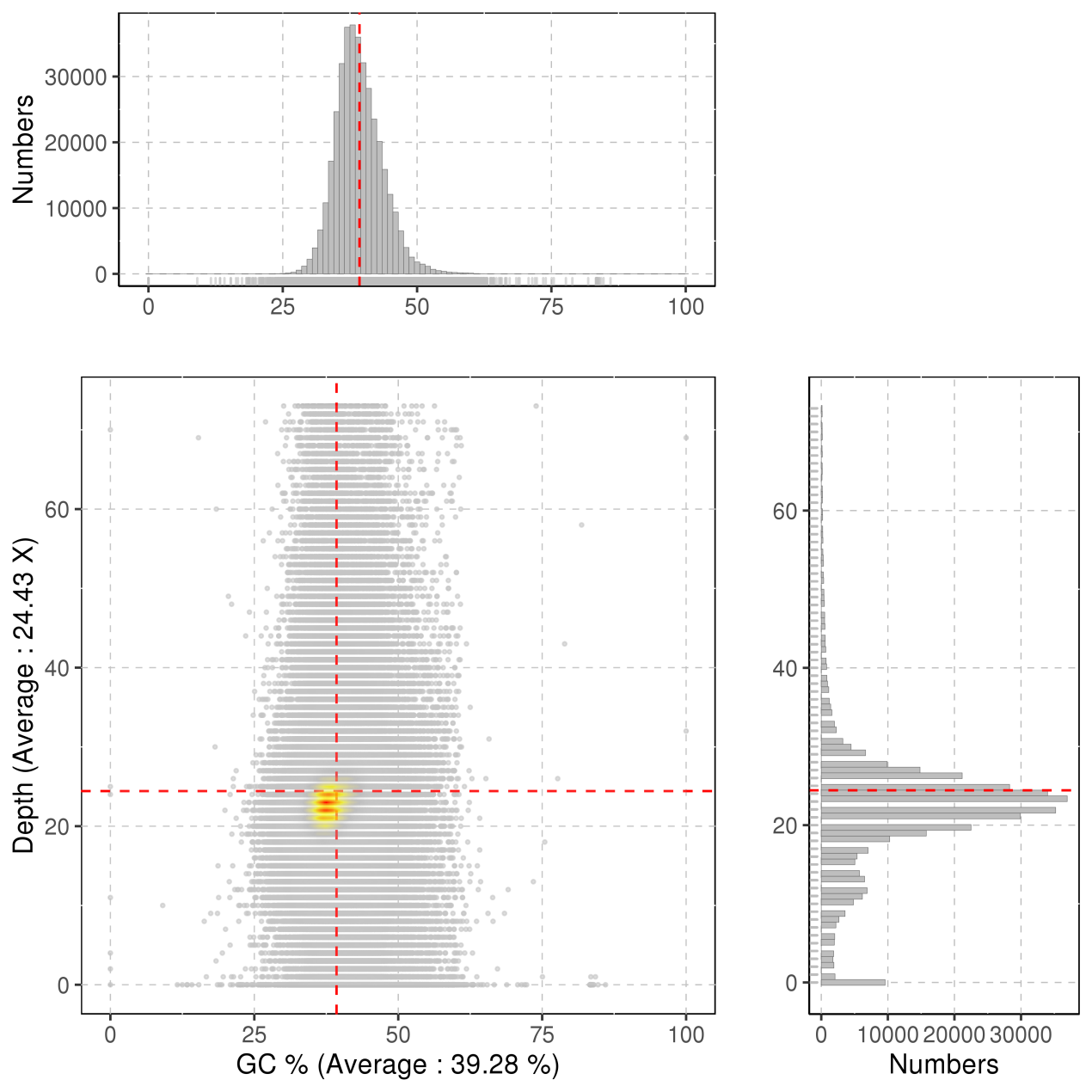
**

**Supplemental** **Figure 5. The GC content distribution of the *M. tetraphylla* genome.**

**
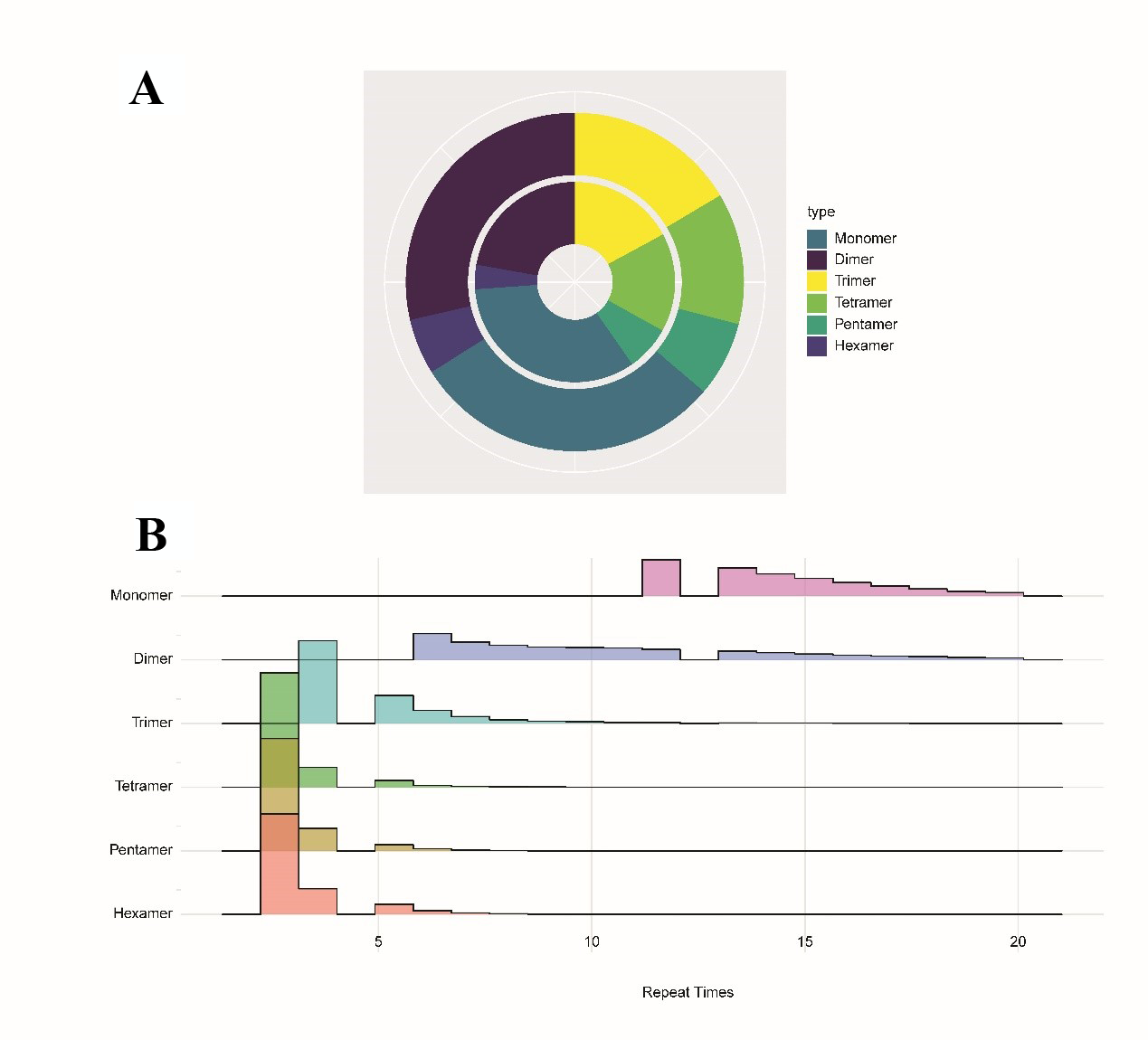
**

**Supplemental** **Figure 6. Summary of SSRs detected in *M. tetraphylla* genome. (A)** Proportion of different types of SSRs. The outer circle represents the length proportion and the inner circle represents the number proportion; **(B)** Repeat times distribution of SSRs.


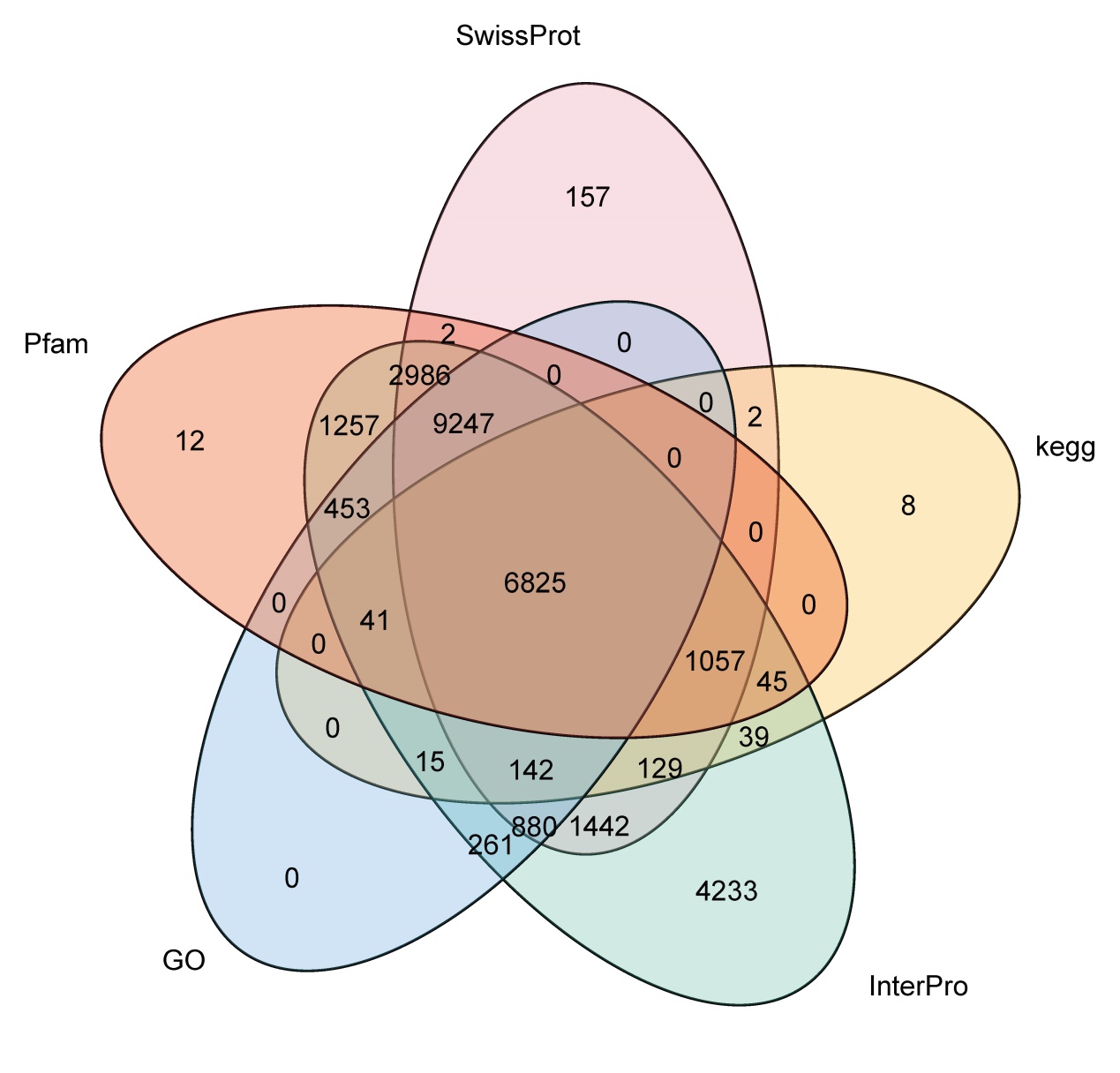


**Supplemental** **Figure 7. Venn diagram of functional annotation.**

**
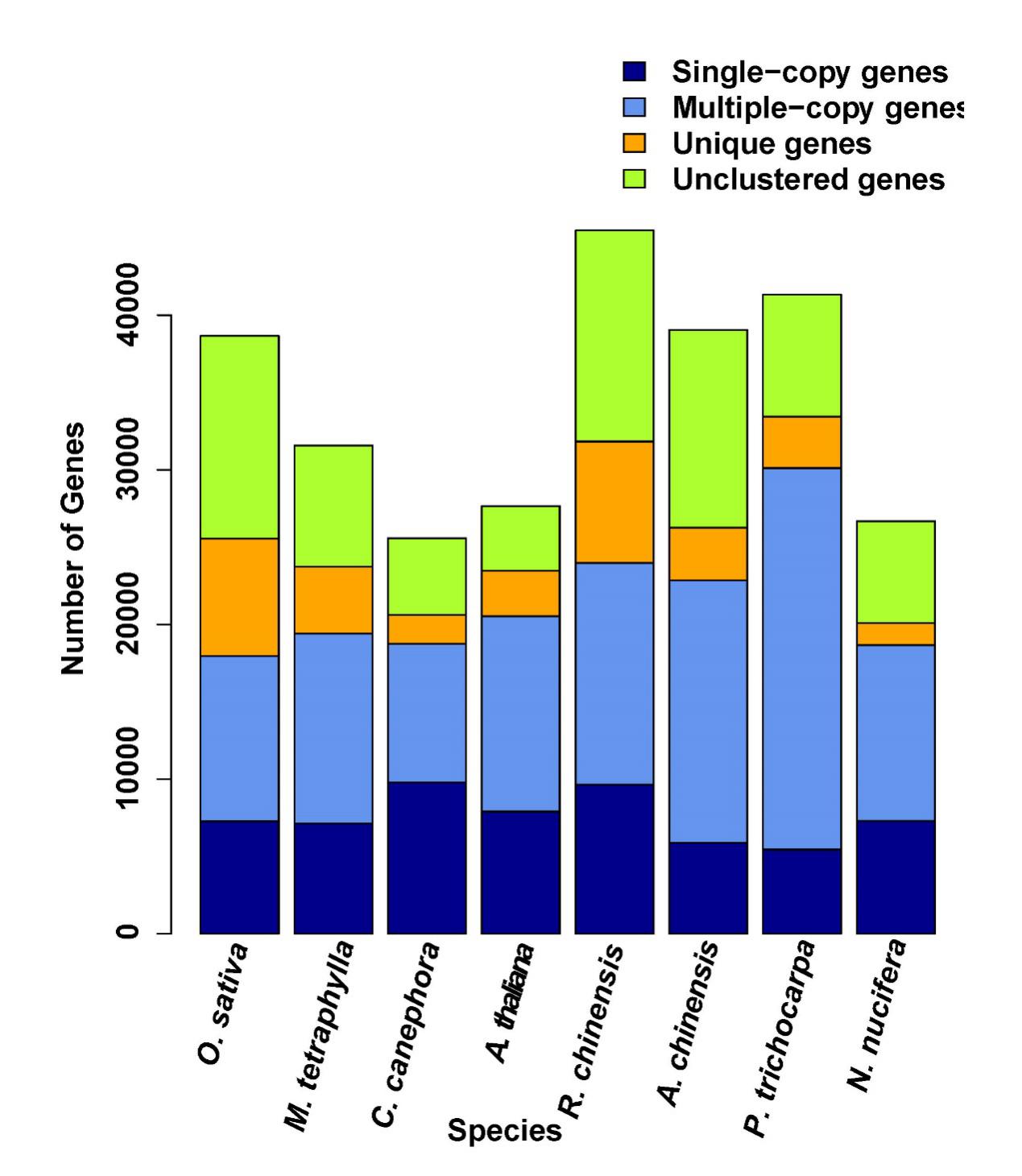
**

**Supplemental Figure 8.** **Distribution of genes and gene families of eight plant species we investigated.**

**
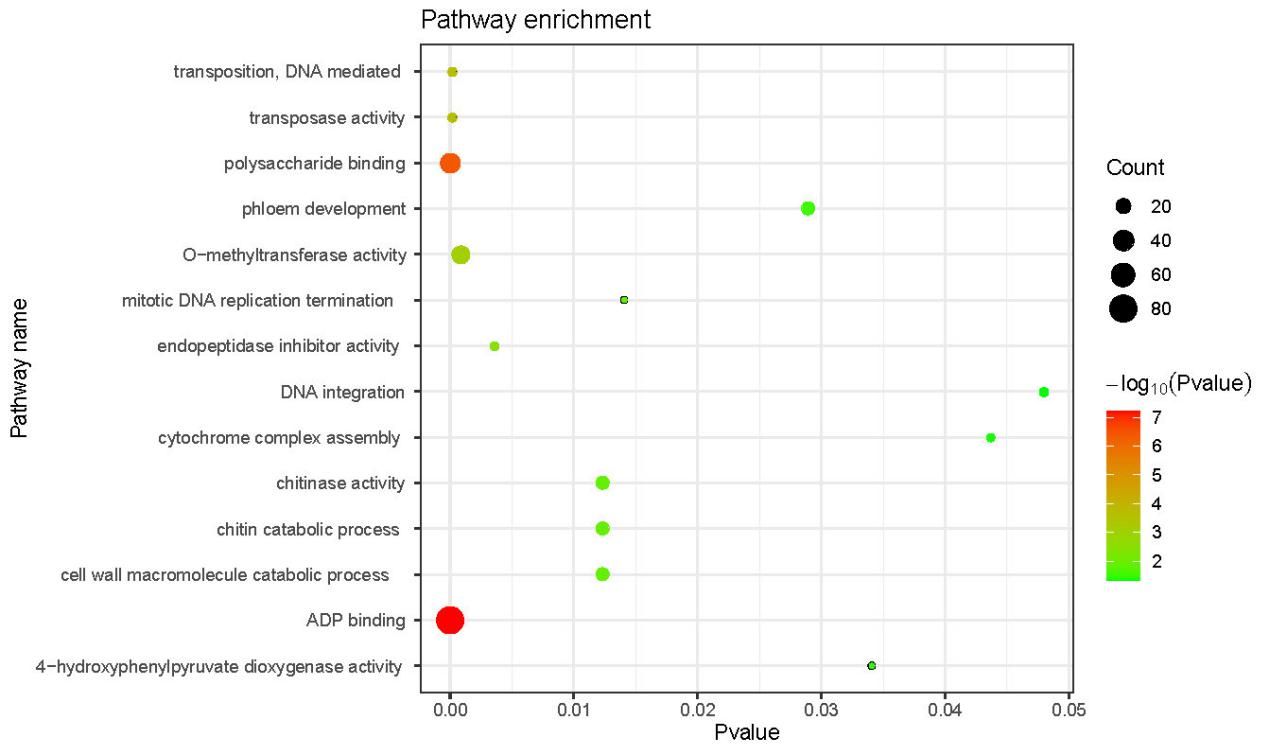
**

**Supplemental Figure 9.** **GO analysis of unique gene families in the macadamia genome.**

**
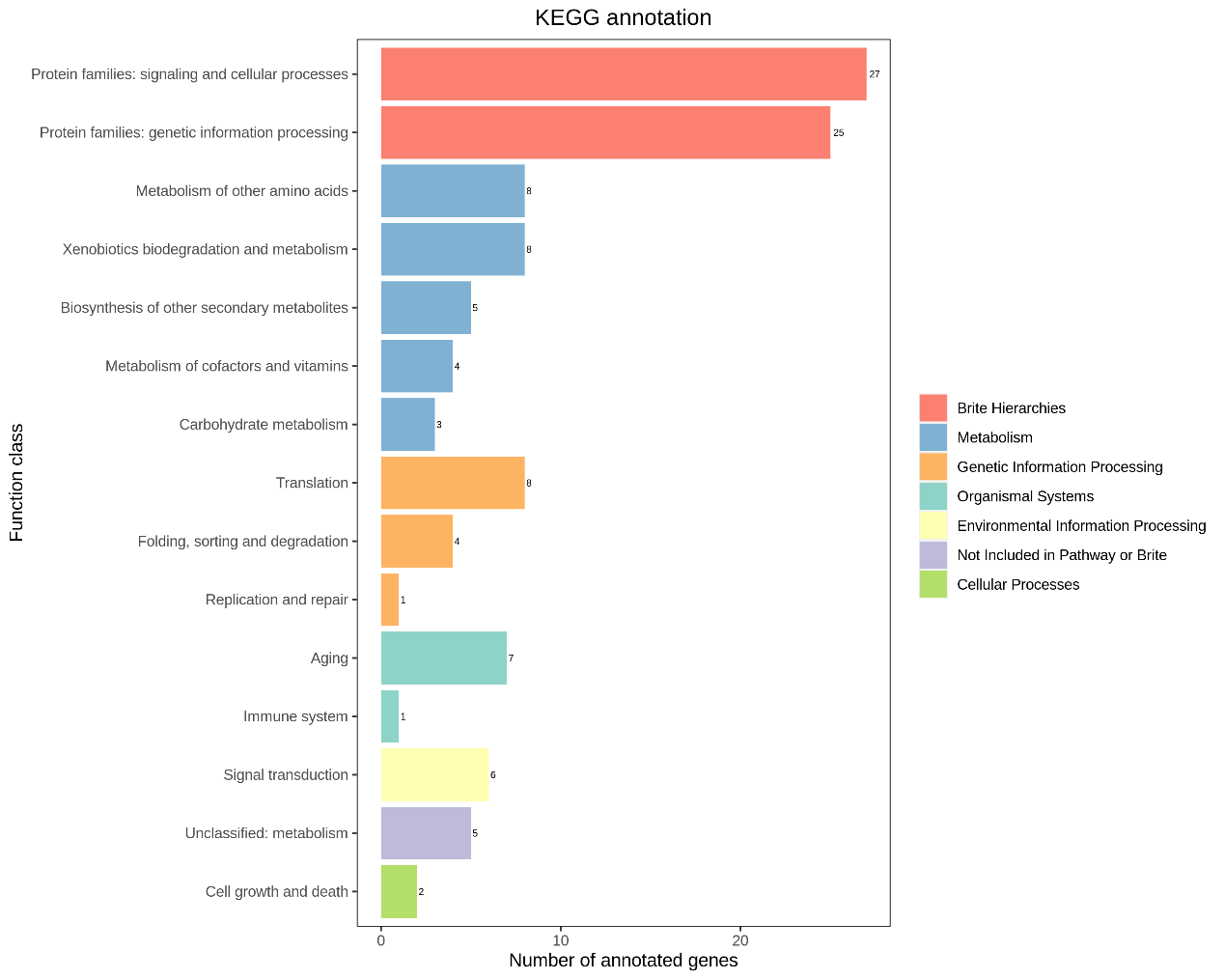
**

**Supplemental Figure 10.** **KEGG pathway annotation of unique gene families in the macadamia genome.**

**
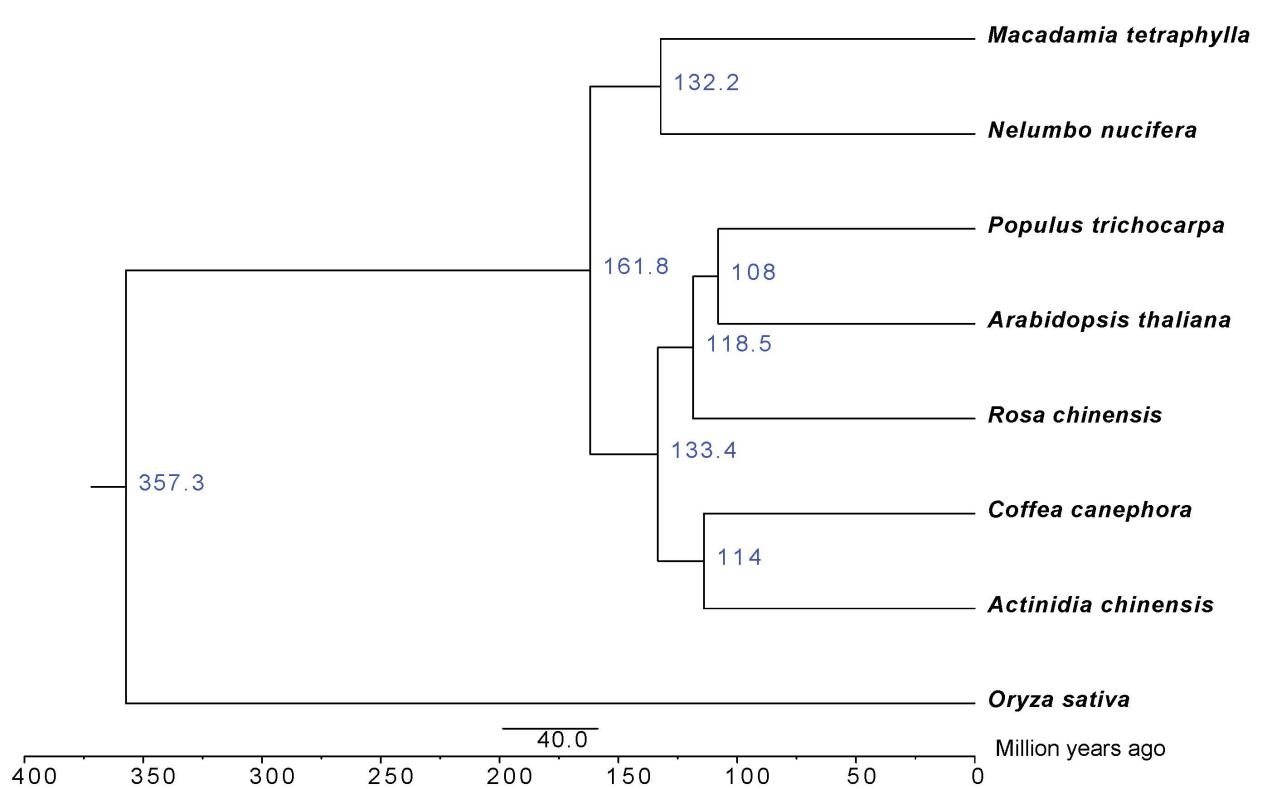
**

**Supplemental Figure 11. Phylogenetic relationships and divergence times of eight plant species investigated in the present study.**

**
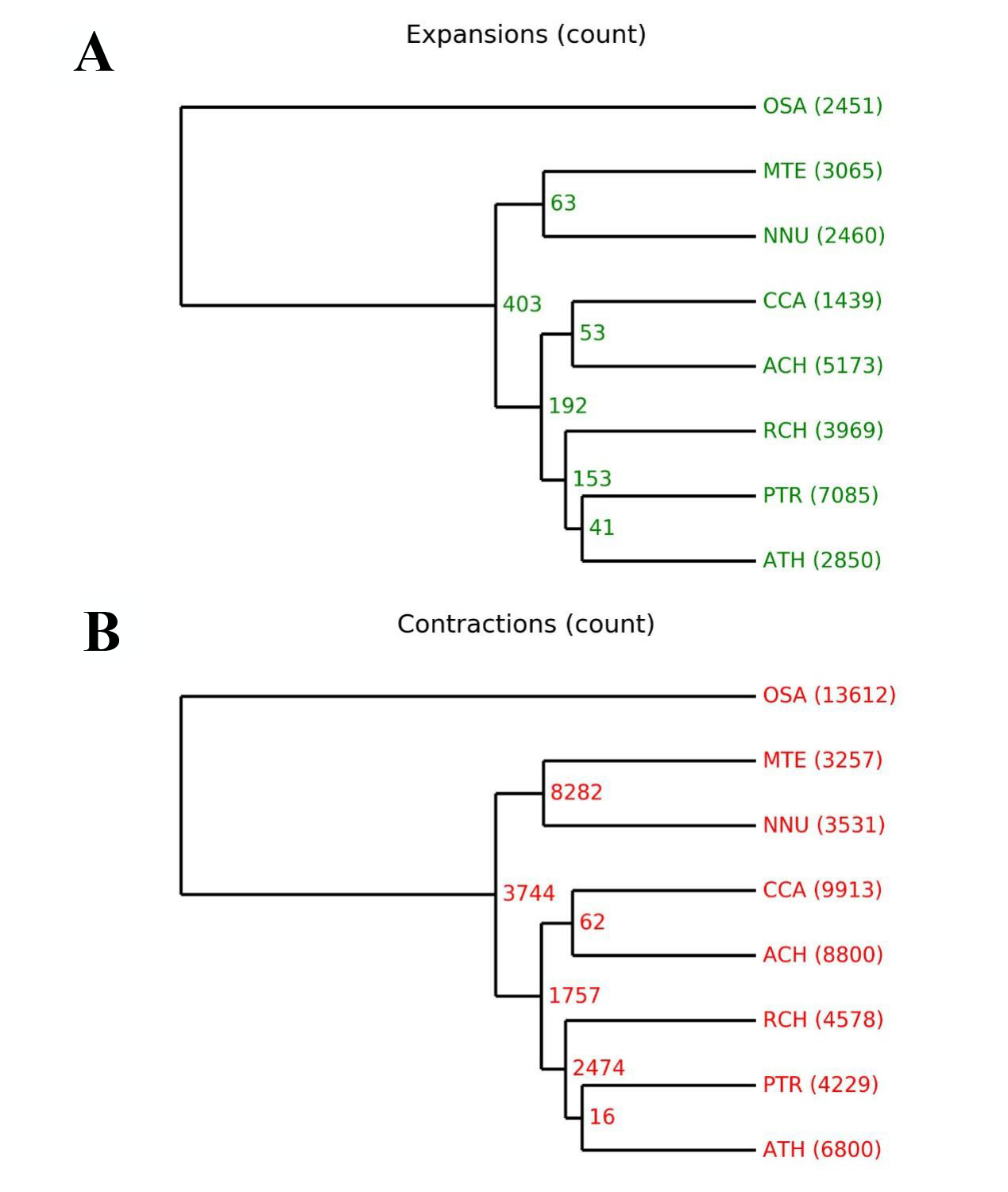
**

**Supplemental Figure 12. Summary of gene families underwent expansion (A) and contraction (B).** ACH, *Actinidia chinensis*; ATH, *Arabidopsis thaliana*; CCA, *Coffea canephora*; NNU, *Nelumbo nucifera*; OSA, *Oryza sativa*; PTR, *Populus trichocarpa*, RCH, *Rosa chinensis*
